# Transcriptomic Analysis of a Susceptible African Maize Line to *Fusarium verticillioides* Infection

**DOI:** 10.3390/plants9091112

**Published:** 2020-08-28

**Authors:** Humaira Lambarey, Naadirah Moola, Amy Veenstra, Shane Murray, Mohamed Suhail Rafudeen

**Affiliations:** 1 Department of Molecular and Cell Biology, University of Cape Town, Private Bag, Rondebosch 7701, South Africa; hlambarey@gmail.com (H.L.); naadirahmoola@gmail.com (N.M.); Amy.Veenstra@alumni.uct.ac.za (A.V.); 2 Center for Proteomic and Genomic Research, Observatory, Cape Town 7925, South Africa; shane.murray@cpgr.org.za

**Keywords:** maize transcriptome, *Fusarium verticillioides*, phytoalexins, kauralexins

## Abstract

Maize (*Zea mays* L.) is a staple crop providing food security to millions of people in sub Saharan Africa. *Fusarium verticillioides*, an important fungal pathogen, infects maize causing ‘*Fusarium* Ear Rot’ disease, which decreases maize kernel yield and the quality of the crop harvested. Currently, no African maize line is completely resistant to infection by *F. verticillioides*. This study investigated an African maize line, *Zea mays* CML144, infected with *F. verticillioides*. Analysis of morphological characteristics showed significant differences between mock-infected and infected plants. RNA-sequencing (RNA-seq) was conducted on plants 14 days post-inoculation to identify differentially expressed genes (DEGs) involved in *F. verticillioides* infection. Analysis of RNA-seq data revealed DEGs that were both significantly up- and down-regulated in the infected samples compared to the mock-infected control. The maize *TPS1* and *cytochrome P450* genes were up-regulated, suggesting that kauralexins were involved in the CML144 defense response. This was substantiated by kauralexin analyses, which showed that kauralexins, belonging to class A and B, accumulated in infected maize tissue. Gene ontology terms relating to response to stimulus, chemical stimulus and carbohydrate metabolic processes were enriched, and the genes belonging to these GO-terms were down-regulated. Quantitative real-time PCR was performed on selected DEGs and measurement of phytoalexin accumulation validated the RNA-seq data and GO-analysis results. A comparison of DEGs from this study to DEGs found in *F. verticillioides* (ITEM 1744) infected susceptible (CO354) and resistant (CO441) maize genotypes in a previous study, matched 18 DEGs with 17 up-regulated and one down-regulated, respectively. This is the first transcriptomic study on the African maize line, CML144, in response to *F. verticillioides* infection.

## 1. Introduction

Maize (*Zea mays* L.) is important in the diets of many people in sub-Saharan Africa [1,2,3]. Currently, it is the third most traded crop worldwide after wheat and rice in terms of its consumption [4]. In South Africa, approximately 8 million tons of maize is produced annually with two-thirds being consumed domestically [5,6].

Maize is susceptible to a variety of fungal pathogens, including, but not limited to, *Alternaria*, *Aspergillus*, *Bipolaris* and *Fusarium* spp. [1,7]. *Fusarium verticillioides* is a common fungal species isolated from maize crops [1,8], and can result in *Fusarium* ear rot (FER) disease, which reduces grain yield and grain quality [9,10,11]. More importantly, *F. verticillioides* infection can also lead to the production of toxic fumonisins, which are associated with cancers and birth defects in humans [2,12]. These toxins are also a health concern to animals where they have been associated with a range of diseases [1,12,13,14]. This renders the crop unsuitable for consumption by both humans and livestock and negatively affects national food security, food safety and agronomy [1].

At present, there are no maize cultivars in southern Africa that are completely resistant to FER [3]. It has been suggested that there are two components important for resistance to *Fusarium* infection; the resistance of the plant to initial penetration (by the pathogen) and prevention of the spread of the pathogen in the plant tissue [15]. Rose et al. (2017) evaluated the resistance of 10 inbred African maize lines known to be resistant to: *Aspergillus* ear rot coupled with aflatoxin accumulation and FER coupled with fumonisin accumulation. Results of this study indicated that these inbred resistant lines showed possible resistance to FER and could be a source of genes for creating FER-resistant maize lines [11].

Analyses of the maize transcriptome to fungal infection is important as it allows for the identification of genes and biological processes involved in the maize defence response [16]. Next-generation RNA-sequencing has become the preferred method in transcriptomic studies, especially in the understanding of plant–fungal interactions [17,18,19,20]. It provides a platform to view and quantify gene expression at the transcriptional level with great detail and allows relevant plant biological processes involved in defense to be highlighted in response to infection [21]. 

Lanubile et al. [22] conducted an RNA-seq study observing the transcriptional changes associated with *F. verticillioides* infection of a resistant (CO441) and susceptible (CO354) maize genotype. The response to infection in both genotypes included: the activation of genes involved in pathogen recognition, signalling and defence (including jasmonate/ethylene mediated responses), with a greater induction in the resistant line. The expression of genes included WRKY transcription factors and were associated with synthesis of phytohormones, phytoalexins and other secondary metabolites [22]. In another study, the maize terpenoid phytoalexins namely, kauralexins and zealexins, had greater accumulation in the moderately FER-resistant maize line, CML444, as compared to the FER-sensitive maize line, CML144, indicating their importance in providing resistance to FER [23]. Additionally, reduced expression of a key kauralexin biosynthetic gene, *ZmAn2*, in an *an2* maize mutant, resulted in greater susceptibility to FER infection as compared to the wild-type [24].The maize kauralexin biosynthetic pathway has been further explored using a systems biology approach where it was shown that kauralexin biosynthesis proceeds via the positional isomer ent-isokaurene [25]. In brief, *ZmAn2* catalyses the cyclization of geranylgeranyldiphosphate (GGPP) to ent-copalyl diphosphate (ent-CPP) and *ZmKSL2* sequentially converts the ent-CPP to ent-isokaurene, which then enters the kauralexin biosynthesis pathway. Co-expression of a cytochrome P450 (ZmCYP71Z18) with *ZmAn2* and *ZmKSL2* demonstrated that there was oxidation of ent-isokaurene to KB1 while *ZmKR2* enables the production of class-A kauralexins (A1, A2 and A3) from the B-class kauralexins precursors (B1, B2 and B3) with ent-kaurene oxidases (*ZmKO2*) enabling the synthesis [25]. *ZmTPS1* also converts ent-CPP to ent-kaurene, which is either converted to the kauralexin precursor KA1 via Z16/18 and/or serves as the central precursor of the gibberellin (GA) pathway [25]. ent-CPP is also converted into dolabradiene by *ZmKSL4* (GRMZM2G016922) and subsequently 15,16-epoxydolabrene (epoxydola- brene) and epoxydolabranol via sequential C-16 epoxidation and C-3 hydroxylation by the cytochrome P450, ZmCYP71Z16 (GRMZM2G067591). Collectively, these labdane-related diterpenoids are termed dolabralexins and together with the ent-kaurene-derived kauralexins are part of the fungal-elicited ent-copalyl diphosphate synthase (ent-CPS) pathway [26].

To our knowledge, there are only a few studies [1,11,12] on the interaction of *F. verticillioides* with African maize lines. As such, it must be noted that RNA-seq studies of maize–*F. verticillioides* interactions are challenging to compare across studies given the differences in the method of infection, maize genotypes, maize tissue used for analyses, field versus plant growth rooms, and other variables [22,27,28,29].

In this study, an RNA-seq approach was used to characterise the interaction between an African maize line, CML144, susceptible to ear rot [30] to *F. verticillioides* infection. The soak-seed method was used for infection and the maize shoot transcriptome analysed 14 days post-inoculation. The aim of this study was to investigate key maize defence genes and responses to *F. verticillioides* infection in order to facilitate future breeding or transgenic approaches towards African maize lines resistant to *Fusarium*.

## 2. Results and Discussions

### 2.1. Plant Morphology and Fungal Quantification

**Figure 1:**
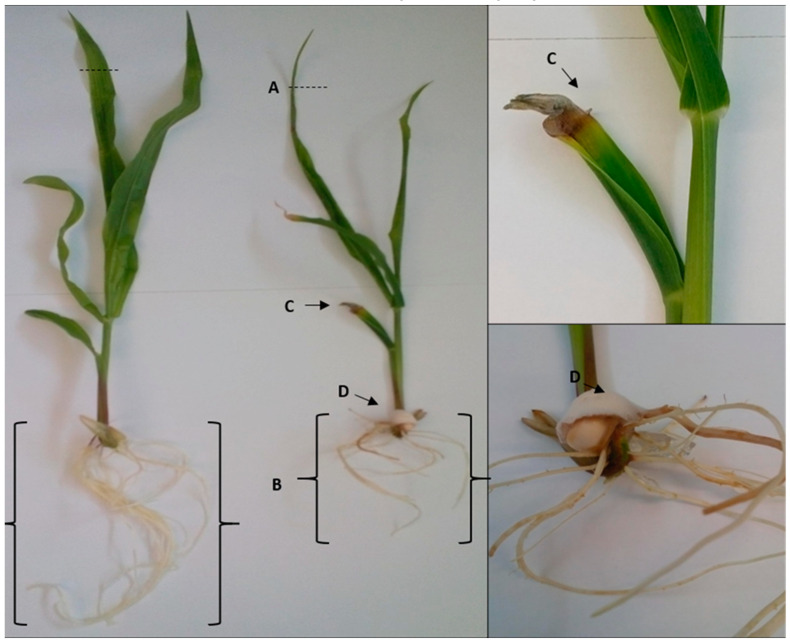
Maize control and infected plants after growth in MS media. Phenotypic differences of control (**left**) and infected (**right**) maize plants showing the leaves, stems and roots after 2 weeks of growth in MS media with distinct differences observed between the control and infected groups. The following were observed for the maize plants infected with *F. verticillioides* when compared to the control: (**A**) inward leaf curling, (**B**) shorter root length, (**C**) superficial fungal growth and discoloration of the fourth leaf, and (**D**) seed covered in fungal growth.

The morphological characteristics of maize plants were observed 14-days post-inoculation to determine whether infection with *F. verticillioides* had any effect on the growth of the plants. Both control and infected plants (grown in MS media) had three to four leaves attached, which showed signs of colour loss and wilting after the 2 weeks of growth. However, distinct differences were also observed between the control and infected plants where the infected plants were stunted with leaves starting to curl inwards (Figure 1). Furthermore, infected leaves also had fungal growth on the fourth leaf and had roots that were shorter with visible signs of rotting (Figure 1).

A final concentration of 1 × 10^3^ conidiospores/mL was chosen after a range of concentrations were tested over a period of 7 to 21 days [data not shown] with the 14-day growth period chosen as optimal. By observing the phenotypical characteristics of the plants, an inoculum greater than concentration 1 × 10^5^ conidiospores/mL resulted in poor or no germination of the maize kernels with the entire seed covered in fungal growth. Growing the plants for longer than 14 days resulted in complete wilting and ultimately death of the infected plant under these conditions.

In planta fungal quantification [31] was performed on control and infected maize shoots to determine the amount of total fungal DNA present (no discrimination between DNA of living or dead fungi). No fungal DNA was detected in control shoots with variable fungal accumulation across the four infected plants at an average of 3.06 ng *F. verticillioides* DNA per μg of maize DNA (Table S3). It was previously shown that *F. verticillioides* DNA isolated from CML144 shoots was shown to be approximately 10-fold lower than in the roots at the same point after inoculation [23].

### 2.2. Production of Antioxidant Enzymes in Maize Shoots in Response to F. verticillioides Inoculation

**Figure 2:**
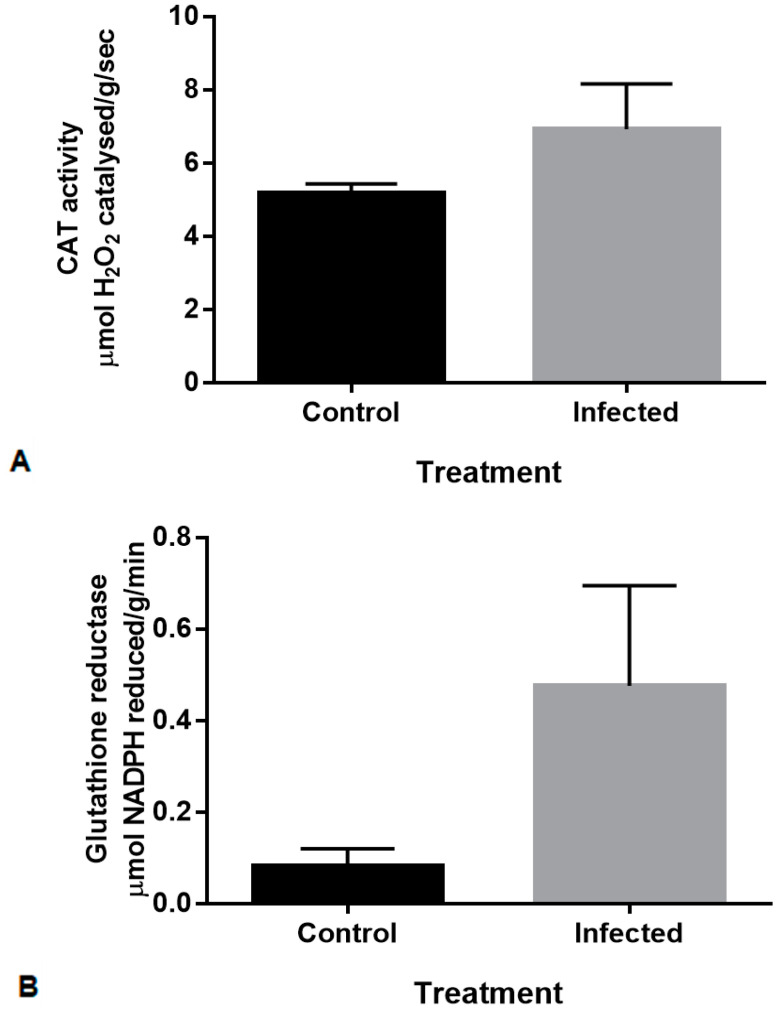
Antioxidant activity in control and infected maize shoots 14 days post inoculation with *F. verticillioides*. (**A**) Catalase (CAT) activity displayed as specific activity (µmol H_2_O_2_ catalysed/g/sec) and measured at a wavelength of 240 nm. (**B**) Glutathione reductase (GR) displayed as specific activity (µmol NADPH reduced/g/min) and measured at a wavelength of 340 nm. The assay was performed on three biological replicates (*n* = 3) from both control and infected samples. Error bars indicate standard error of the mean (SEM).

Physiological assays were conducted to determine whether infection of the plants resulted in an antioxidant response. Previous analysis of maize lines that were either resistant or susceptible to FER indicated that antioxidant enzymes were produced in kernels in response to inoculation with *Fusarium* spp. [32]. In order to establish whether maize shoots displayed similar responses, the enzymatic activity of superoxide dismutase (SOD), glutathione reductase (GR) and catalase (CAT) were measured in maize shoots 14-days post inoculation with *F. verticillioides* (Figure 2). Enzyme isoforms were not distinguished in these assays.

The SOD assay showed no difference between the control and infected and was not statistically significant [data not shown]. An increase in enzyme activity following *F. verticillioides* inoculation can be seen for CAT (Figure 2A) and GR (Figure 2B), although these were not statistically significantly when compared to the controls (*p* values = 0.22 and 0.08, respectively). This is possibly due to biological variation in the activity of the respective antioxidants measured within the individual samples where each biological has differences in quantitative and qualitative antioxidant responses to pathogen attack. In addition, these enzymes are expressed at different stages of plant growth and at different locations. For CAT activity in maize, three catalase isoforms exist and are encoded on different chromosomes. These isoforms have a high sequence similarity at both the amino acid and nucleotide levels [33,34]. It has been shown that these isoforms have distinct patterns of transcript and protein accumulation [35]. Additionally, SOD and GR also have several isoforms that are located in different regions of maize leaves [36], these differences could account for the variability in the results. Therefore, the high levels of CAT (Figure 2A) in the control shoots could be isoform-specific, genotype-specific, or seedling growth-stage-dependent, due to developmental changes or a result of in situ growth conditions.

It is important to note that the level of antioxidant enzyme activity induced by different *Fusarium* spp. is variable [32]. Lanubile et al. [37] reported that in response to *F. verticillioides* (ITEM 1744) infection, SOD activity in infected tissue was higher and CAT activity in infected tissue was lower in the resistant compared to the susceptible maize line. However, in terms of a general response in the susceptible line, an increase was seen for both SOD and CAT activity in the infected tissue compared to the control tissue [37]. Additionally, the same maize line may respond differently to infection with various fungal pathogens [32,37]. Lanubile et al. [32] determined the enzyme activity in maize kernels to three different fungal pathogens, which included *F. proliferatum*, *F. subglutinans* and *Aspergillus flavus*. CAT activity increased in response to these pathogens, whereas SOD activity decreased. GR activity showed no difference in response to all three pathogens [32]. Comparing our results (conducted on susceptible maize shoots) to that of Lanubile et al. [32,37] (conducted in maize kernels), the antioxidant response of CAT was similar despite the differences in tissue used and analysed. Kumar et al. [38] found an increase in CAT, SOD and GR activity in both the roots and shoots in response to *F. verticillioides* infection. This suggests that the various antioxidant responses in maize to various fungal pathogens are modulated depending on the tissue type, maize line and mode of infection by the fungal pathogen.

Lastly, the enzyme assays used in the current study do not distinguish between isoforms and it is possible that at the time the assays were conducted, specific enzyme isoforms were expressed in a different region of the plant or were functionally inactive. 

### 2.3. Determining Differential Expression between Control and Infected Maize Shoots Using RNA-Sequencing 

With the changes seen in the morphology of infected maize leaf and root, increased *F. verticillioides* biomass after infection and differences in maize antioxidant responses; whole transcriptome RNA-seq was used to investigate changes in the corresponding gene expression. The gene expression results from the RNA-seq data obtained in this study were analysed in DNA Subway (Protocol 1, see materials and methods), and a second protocol was conducted at the CPGR (Protocol 2, see materials and methods). 

Of all the reads mapped to the B73 v2 genome using Protocol 1, there were a total of 128 candidate genes found to be significantly differentially expressed in the infected group compared to the control group (*q* < 0.01). Of these 128 candidate genes, 53 were down-regulated and 75 were up-regulated, respectively (Tables S5 and S6). 

**Figure 3:**
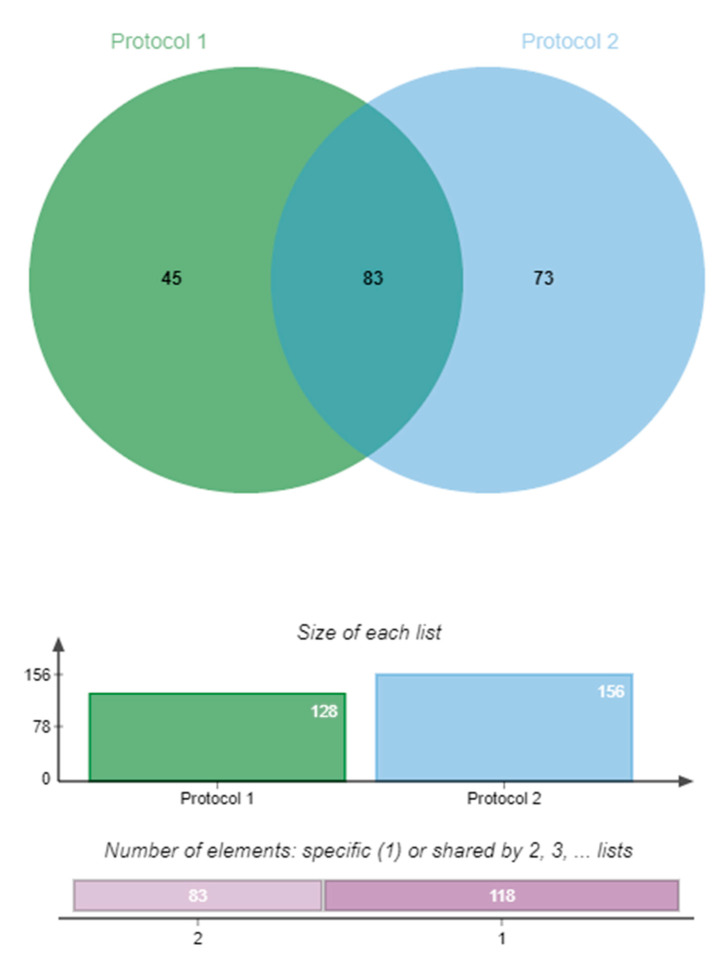
Venn diagram showing the total DEGs that were up-regulated, down-regulated and commonly expressed between the two protocols. The diagram was created using jvenn [39].

Analysis from Protocol 2 (sequencing reads mapped to the B73 v3 reference genome) showed 156 differentially expressed candidate genes significantly expressed in the infected group compared to the control group (*q* < 0.01). Of these 156 candidate genes, 50 were down-regulated and 106 were up-regulated, respectively (Tables S7 and S8). More genes were found to be differentially expressed using Protocol 2 compared to Protocol 1, possibly due to a difference in the read filtering methods applied and the use of two different versions of the B73 annotated reference genome. However, of the gene matches, both protocols identified the same 54 up-regulated and 29 down-regulated genes (total = 83) (Table 1). A Venn diagram (jvenn [39]) showing the total amount of DEGs that were up-regulated, down-regulated and commonly expressed between the two protocols can be seen in Figure 3.

### 2.4. Gene Ontology Enrichment Analysis Reveals Possible F. verticillioides Induced Response Mechanisms in Maize 

To gain a better insight into the function of the DEGs found by RNA-seq analysis, the matching up- and down-regulated DEGs from Protocol 1 and Protocol 2 (Table 1) were annotated using the online gene ontology analysis tool, agriGO [40]. 

Up-regulated and down-regulated genes were annotated separately using the agriGO database and mapped to the *Zea mays* AGPv3.30 reference genome (Figures S1 and S2). Of the 54 matching genes shown to be up-regulated by RNA-seq analysis, only 45 were annotated in agriGO. Analysis revealed only three significant GO-terms (*p* < 0.05, multi-test adjustment [Yekutieli—FDR under dependency]) as seen in Table 2. These GO terms belonged only to the molecular function (F) GO category. GO-terms relating to cation, ion and metal ion binding were shown to be significantly enriched in the up-regulated genes (Table 1, Table 2, and Figure S1). 

The up-regulated ent-Kaurene synthase B gene (GRMZM2G049538), also called terpene synthase 1 (*TPS1*), was one of the up-regulated genes associated with the three enriched GO-terms, which include: GO:0043169 (cation binding), GO:0043167 (ion binding) and GO:0046872 (metal ion binding) (Table 2). This is important to note because ent-Kaurene is one of the intermediates in the synthesis of gibberellin and kauralexins in both fungi and plants [26,41,42,43]. Interestingly, the cytochrome P450 family expressed gene (GRMZM2G087875), an up-regulated gene found in this study and enriched for all three GO-terms, is putatively involved in the last steps of both gibberellin and kauralexin biosynthesis [26,44,45]. These kauralexins are terpenoid phytoalexins and are associated with the defence response against pathogen attack [46].

Genes associated with antioxidant activity were found within the list of genes associated with these GO-terms. Within the matching up-regulated significant GO-terms, the GRMZM2G427815 gene is associated with peroxidase and oxidoreductase activity. Peroxidase is one of the enzymes involved in scavenging reactive oxygen species (ROS) when the host is under pathogen attack or other stresses [38]. In addition, this gene was also found to be expressed in both the resistant and susceptible genotypes [22]. 

The other genes associated with all three GO-terms are mostly uncharacterised proteins but also include an alcohol dehydrogenase gene (GRMZM2G098346) that is involved in abscisic acid sensitivity, biotic stress resistance as well as sugar accumulation. This gene was studied in *Arabidopsis*, which showed that the alcohol dehydrogenase 1 (*ADH1*) gene conferred resistance to both abiotic and biotic stress [47]. 

An analysis of the down-regulated genes found in the match between Protocol 1 and Protocol 2 resulted in 22 of the 29 genes being annotated. Analysis revealed three significant GO-terms (*p* < 0.05, multi-test adjustment [Yekutieli–FDR under dependency]) as seen in Table 2 (also see Figure S2) with these GO-terms belonging to only the biological process (P) category. GO terms relating to response to stimulus, response to chemical stimulus and carbohydrate metabolic processes were shown to be significantly enriched in the down-regulated genes. The genes belonging to these GO-terms are also involved in metabolic processes, response to abscisic acid stimulus, sucrose and starch catabolic processes, amongst other responses; although some of these GO-terms were not enriched. 

In the response to stimulus and response to chemical stimulus GO-term, several genes (GRMZM2G004161, GRMZM2G070172, GRMZ2G125775, GRMZM2G181081) have child terms that are involved in response to salicyclic, jasmonic and especially abscisic acid stimulus. Salicyclic and jasmonic acid are involved in the biotic stress response, whereas abscisic acid is involved in the abiotic stress response (a negative regulator of disease resistance). The above-mentioned hormones, although involved in different stress responses, are known to occur simultaneously at differing levels [48]. The suppression of these responses would be expected in terms of *F. verticillioides* breaching the defence response by either suppressing the salicyclic acid pathway or modulating the hormone response pathway to favour infection. Studies have shown that in response to *F. oxysporum* infection, *Arabidopsis* mutants deficient in SA-mediated defence were more susceptible to the fungus [49]. In contrast, an induction of JA-mediated defence response was seen in the leaves of *Arabidopsis* during infection with *F. oxysporum* [50]. The GRMZM2G004161 gene has child terms, which include: GO: 0009751(response to salicylic acid stimulus), GO: 0009753 (response to jasmonic acid stimulus), GO: 0009738 (abscisic acid mediated signaling pathway), GO: 0009737 (response to abscisic acid stimulus) and GO:0080134 (regulation of response to stress). Gene GRMZM2G070172 has child terms which include GO: 0009739 (response to gibberellin stimulus) and GO: 0009737 (response to abscisic acid stimulus). Gene GRMZ2G125775 has GO: 0009737 (response to abscisic acid stimulus) and GO: 0010200 (response to chitin) while gene GRMZM2G181081 has GO: 0009737 (response to abscisic acid stimulus) child terms, respectively. This down-regulated response is interesting and suggests that *F. verticillioides* might be modulating plant hormones.

The genes associated with sucrose, glucose and trehalose synthesis in the GO-term carbohydrate metabolic process were also down-regulated. In an RNA-seq study involving arbuscular mycorrhiza and the plants *Lotus japonicas* and *Rhizophagus irregularis*, there were several down-regulated genes that included GO-terms related to carbohydrate metabolism. In this study, fungal colonisation by the arbuscular mycorrhiza led to a decrease in starch, which indicated that the starch was broken down to provide carbohydrates required by the fungus [51]. The RNA-seq study by Wang et al. (2016), which investigated the mechanisms of resistance to *F. verticillioides* by maize, found after enrichment analysis of both up- and down-regulated genes, that GO-terms associated with sucrose and starch metabolism were the most significantly enriched pathways after infection [29]. This information suggests that the fungus drives the plant to make specific carbohydrates as a food source. Campos-Bermudez et al. (2013) also found important changes occurring within pathways related to carbon metabolism in a susceptible maize line but not in a resistant maize line upon *F. verticillioides* infection. They suggest that source cells reprogrammed carbon flow from sucrose to hexoses as part of plant defence, while fungal pathogens manipulate plant carbohydrate metabolism to favour glucose over sucrose [52]. It was shown that amylopectin induces fumonisin B1 biosynthesis as the *F. verticillioides* amylase mutant could not produce the fumonisins on starchy kernels [53]. It is thus possible that the decrease we observe in processes associated with carbohydrate metabolism in our study is due to the sequestering of plant metabolites by *F. verticillioides* or as part of plant defence mechanisms. When pathogens attack maize plants, carbohydrate metabolism is one of the main metabolic processes that are affected. However, an in-depth analysis of the maize–*F. verticillioides* pathosytem relating to sugar metabolism is necessary. This is to determine how the changes in metabolism are linked to disease development, resistance to the fungus and the establishment of the pathogen [9].

Hydrogen peroxide, a ROS molecule produced after pathogen attack, is involved in plant defence response by cross-linking cell walls, direct antimicrobial action against the pathogen, induction of gene expression, the hypersensitive response, phytoalexin production, and induced systemic resistance [33,54]. Within the down-regulated significant GO-terms related to the antioxidant response, the genes GRMZM2G103812 and GRMZM2G004161 are associated with two of the three enriched GO-terms (GO: 0050896 and GO: 0042221) and one other specific child term (GO:0042542), which involves the response to hydrogen peroxide. The downregulation of these genes in response to the presence of hydrogen peroxide could also be a contributing factor to CML144 susceptibility to *F. verticillioides* as plant defence responses may be dampened during infection. However, genes associated with the ROS assays conducted in this study were not differentially expressed nor were they associated with any of the GO-terms within the analysis.

### 2.5. Comparing DEGs Against a Different Maize Line Infected with F. verticillioides

To compare defence responses to *F. verticillioides* (ITEM 1744) infection, the DEGs in this study were compared with the DEGs found in the study by Lanubile et al. [22] where RNA-seq analysis was performed on a susceptible (CO354) and resistant (CO441) maize genotype, respectively. It must be noted that the authors used the pin-bar method of infection and analysed the kernels as compared to the soak-seed method of infection and the analysis of the shoots used in this study. The DEGs found for both the resistant and susceptible genotypes as well as common DEGs (genes found in both the resistant and susceptible genotype) were compared to the DEGs from the current study (Table 3). 

In total, there were 17 up-regulated (Table 3) and one down-regulated DEGs (GRMZM2G181081/ CIPK-like protein 1) that matched differentially expressed up- or down-regulated genes in the Lanubile et al. [22] study. Even though only 17 genes were up-regulated in both studies (Table 3), it also suggests that there could be a particular set of maize genes expressed in response to *F. verticillioides* infection, despite differences in *F. verticillioides* strains, the method of infection, maize lines, sampling times, and tissue harvested. Several genes (Table 3) are related to the functional category of metabolic process (41%) and transport (24%) with other categories including resistance, signal transduction, cell wall, and response to stress. The metabolic process category was the largest portion represented in this list and was also most prevalent in the Lanubile et al. study [22]. Changes in gene expression of these genes belonging to these specific categories indicate that the infection by *F. verticillioides* caused a change in plant metabolism response.

### 2.6. Validation of RNA-Sequencing by Quantitative Real-Time PCR

**Figure 4:**
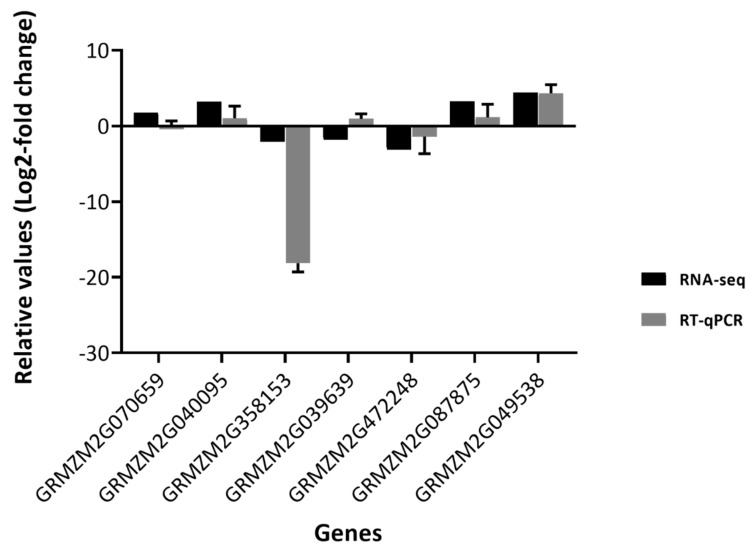
Quantitative real-time PCR analysis of average gene expression (*n* = 3) of selected genes (Table S4) in *F. verticillioides*-infected shoot tissue relative to control shoot tissue 14 days post inoculation. Expression was normalised to the *UBCE, Rpol, MEP* reference genes (Table S4) and shown as relative values (Log2-fold change). The RT-qPCR results were compared against average RNA-seq analysis results (mean of Protocol 1 and Protocol to Log2-fold values). Error bars indicate standard error of the mean (SEM).

Gene expression analysis by RT-qPCR was performed to validate the RNA-seq and GO-analysis results, with DEGs mostly associated with the ‘response to stimulus’ GO-term (GO: 0050896) chosen for analysis. Most of the genes belonging to this GO-term are associated with the defence response, with a ‘response to fungus’ being of specific interest. From the list of DEGs, both up- and down-regulated genes were selected for quantification and expression analysis via qRT-PCR. Genes within our study that were associated with metabolite accumulation were also analysed (Figure 4 and Figure S3).

The following seven genes were selected for RT-qPCR analysis: Hypersensitive induced response protein (ZmHIR3, GRMZM2G07059), Lipoxygenase 6 (Zmlox6, GRMZM2G040095), Chitinase 1 (ZmChitinase 1, GRMZM2G358153), Pathogenesis-related thaumatin-like protein (ZmPR-th, GRMZM2G039639), Protein induced upon tuberization (ZmPIT, GRMZM2G472248), Cytochrome P450 (ZmCYA81A, GRMZM2G087875), and Trehalose-6-Phosphate Synthase 1 (ZmTPS1, GRMZM2G049538). The results for the RT-qPCR experiments were then compared against RNA-seq fold-changes (Figure 4).

The RT-qPCR expression fold-change results were very similar when compared to the RNA-seq analysis results. In the RNA-seq analysis, the GRMZM2G040095, GRMZM2G087875, GRMZM2G049538, and GRMZM2G070659 were shown to be up-regulated; a similar trend was seen with the fold-changes for RT-qPCR (Figure 4). The GRMZM2G039639, GRMZM2G472248, and GRMZM2G358153 genes were shown to be down-regulated during the RNA-seq analysis, and again the same trend was seen with the RT-qPCR results apart from GRMZM2G039639, where there was an increase in fold-change. This difference could possibly be due to variability within the biological replicates or the relative sensitivity of RT-qPCR compared to RNA-seq. The overall RT-qPCR results of the selected genes, although not statistically significant (*p* > 0.05 for all genes), displayed trends that suggest the RNA-seq data and GO analyses may be correlated.

In addition, in a microarray study conducted by Lanubile et al. [27,37], other homologues of *chitinase*, PR and *HIR* genes (pathogenesis-related genes) were shown to be induced in response to *F. verticillioides* infection. These genes are associated with disease resistance and it is possible that these genes are the same as the genes found in our study. However, they could also be different paralogues of the same genes or the same genes found on different chromosomes. The genes in our study could not be confirmed as being the same as those found by Lanubile et al. [27,37], since this study observed changes in gene expression using microarray analysis.

### 2.7. Analysis of Phytoalexin Accumulation 

*ZmTPS1* (GRMZM2G049538) was the mosthighly up-regulated gene in this study as revealed by RNA-seq analysis and is proposed to be a downstream kauralexin biosynthetic gene [26]. *ZmTPS1* synthesizes ent-kaurene from ent-CPP, where ent-kaurene is then synthesized to the kauralexin precursor, KA1 via Z16/18 [25]. For this reason, kauralexin accumulation in maize tissue was also analysed to determine whether artificial infection of the maize with *F. verticillioides* was associated with changes seen in the plants after infection (Table 4). The ent-kaurene synthesized via *ZmTPS1* is also the central precursor of the gibberellin (GA) pathway, which is crucial to plant development and the pathway serves as a target in pathogenesis to favour infection [55].

Metabolites KA3 and KB3, which were significantly increased (Table 4), have previously been shown to directly inhibit fungal growth as well as reduce herbivory in plants [43]. The KA3 metabolite is highly associated with fungal protection in diverse commercial hybrids [56] and underlie *Fusarium* spp. resistance [25]. Metabolite analysis by Vaughn et al. [46] showed that roots infected with *F. verticillioides* contained greater kauralexin accumulation compared to the control roots. This supported the fact that phytoalexins are produced in response to biotic stress in both roots and shoots [43,46].

*ZmAn2* (GRMZM2G044481) is crucial in kauralexin biosynthesis as it catalyses the cyclization of geranylgeranyldiphosphate (GGPP) to ent-copalyl diphosphate (ent-CPP), which is converted sequentially to ent-isokaurene via *ZmKSL2* and is a precursor of the kauralexin pathway and forms the respective KB and KA compounds [25]. The ent-isokaurene is also converted into dolabradiene by *ZmKSL4* (GRMZM2G016922), which has been shown to inhibit both *F. verticillioides* and *F. graminearum* [57].

Interestingly, the *ZmAn2* and *ZmKSL4* genes were not significantly differentially expressed in our respective RNA-seq study and RT-qPCR (Figure S3). It was previously shown that the *an2* maize mutant had increased susceptibility to *F. verticillioides* infection compared to the wild-type [23,24]. It is also noteworthy that the *an2* mutant is deficient in both dolabralexins and kauralexins, indicating that *ZmAn2* provides ent-CPP for both families of labdane-related diterpenoid phytoalexins [46,57,58].

The accumulation of kauralexins in maize tissue as well as the validation of kauralexin-associated genes by RT-qPCR confirmed that the infection of the maize using the soak-seed method was associated with the physiological and transcriptomic changes seen in the plants after the 2 weeks of infection.

## 3. Conclusions

In order to understand the changes in gene expression in maize shoots after *F. verticillioides* infection, an RNA-seq study was conducted to determine DEGs in the susceptible African maize line CML144. Although a number of RNA-seq studies have been done on maize in response to *F. verticillioides* infection, this study was performed on an African maize line using the soak-seed method. Furthermore, this study highlighted the differences in shoot tissue only.

An initial analysis (before RNA-seq) of physiological, biochemical and morphological characteristics of the plants was conducted to ensure successful infection and growth parameters. The biochemical assays focused on SOD, CAT and GR antioxidant enzymatic activity measured 14 days post inoculation. Although a difference in antioxidant activity was seen between the control and infected plants, it was not statistically significant. However, these antioxidant enzymes have many functionally active or inactive isoforms and are known to be differentially regulated during growth stages and in different tissues [33]. Future studies conducted on antioxidant activity may benefit from analysing and differentiating the isoforms of SOD, CAT and GR.

Given the sets of matching up- and down-regulated DEGs (found between Protocol 1 and 2 of the RNA-seq analysis), GO analysis was performed in agriGO, which found significantly enriched GO-terms belonging to molecular function and biological process categories, respectively. GO terms relating to cation, ion and metal ion binding were significantly enriched in the up-regulated genes whereas GO-terms relating to response to stimulus, response to chemical stimulus and carbohydrate metabolic processes were significantly enriched in the down-regulated genes. The identified genes, both up-regulated (GRMZM2G049538, GRMZM2G087875, GRMZM2G427815, GRMZM2G098346) and down-regulated (GRMZM2G004161, GRMZM2G070172, GRMZM2G125775, GRMZM2G181081, GRMZM2G103812, GRMZM2G004161) are associated with the defense response. This suggests that though a maize defense response is mounted in response to *F. verticillioides* infection, it is not sufficient to prevent *Fusarium* infection. In addition, *Fusarium* also modulates the defence response by down-regulating relevant genes and/or pathways. 

The 17 up-regulated genes overlapping between the genes found by Lanubile et al. [22] suggest the possibility of a common gene expression profile across both maize genotypes and tissue types. Notably, RNA-seq also identified *ZmTPS1* as the most significantly up-regulated gene as a defence response in shoot tissue; kauralexins analysis confirmed the accumulation of this secondary metabolite.

We also observed that in the FER-susceptible CML144 maize line, *F. verticillioides* infection did not lead to a notable increase in *ZmAn2* and *ZmKSL4* gene expression, thereby limiting kauralexins and dolabralexins accumulation and consequent protection. In contrast, the up-regulation of *ZmTPS1* upon infection could provide possible pathogen targets for *F. verticillioides* in the GA pathway in preference to shunting ent-kaurene into the kauralexin pathway via synthesis of KA1. Taking these observations together could explain, in part, why this maize line is more susceptible to FER infection.

In conclusion, the changes in gene expression after *F. verticillioides* infection by whole-transcriptome RNA-seq in this study serve as a base for future studies to help broaden our understanding of the maize-*F. verticillioides pathosystem,* and ultimately, developing strategies to produce maize lines resistant to this pathogen.

## 4. Materials and Methods

### 4.1. Fungi

The *F. verticillioides* (Sacc. Nirenberg) strain, MRC 826, used in this study, was provided by Pannar Seed (Pty) Ltd. (Greytown, South Africa). The fungus was maintained at 30 °C and sub-cultured weekly on Potato Dextrose Agar (PDA) (Lab M Limited, Ayr, UK) or stored in 20% glycerol at −70 °C.

### 4.2. Plant Growth, Inoculations and Morphology

Sterilised CML144 maize seeds (*Zea mays* L.) were infected with *F. verticillioides* via artificial inoculation according to Oren et al. [59] with minor modifications. For inoculation, the PDA plates containing the 6–7-day-old fungus were used to isolate 1 × 10^3^ conidiospores/mL for infection in a 2% (*v/v*) Tween20 solution. Seeds to be infected were inoculated in the spore/Tween20 suspension while seeds for the mock-infected (control) were suspended in 2% Tween20 solution only; after which both treatments were incubated at 30 °C for 30 min with shaking. Seeds (control and infected) were dried and then placed on Murashige and Skoog (MS) (Highveld biological (Pty) Ltd, South Africa) media under sterile conditions. Glass jars containing the seeds were incubated in controlled conditions at 28 °C (16 h light, 8 h dark; light intensity of 140 µmol/m^2^/s and 60% humidity) for a period of 14 days (maize V3 leaf stage).

The morphology of the maize plants (control and infected) was observed after the 14 days of incubation. 

### 4.3. RNA Extraction and RNA-Sequencing

At the maize V3 leaf stage, control and infected maize leaves were harvested in liquid nitrogen and RNA was extracted from 500 mg of tissue using TRI Reagent (Zymo Research, USA, CA, Irvine). Each RNA extraction sample was DNase treated as per manufacturer’s instructions (Thermo Fisher Scientific, MA, Waltham) and incubated for 1 h at 37 °C. The samples were then purified using equal volumes of phenol: chloroform-isoamyl alcohol (25:24:1). The RNA was quantified, and the integrity was assessed on a 1.2% EtBr stained agarose gel by electrophoresis. The RNA was further analysed on a Qubit 2.0 fluorometer and on an RNA-6000 Nano chip using the Agilent 2100 BioAnalyzer (Santa Clara, CA, USA).

### 4.4. RNA-Sequencing and Data Analysis

A total of six maize RNA samples isolated from control (3) and infected (3) plants were submitted for RNA-seq at the Centre for Proteomic and Genomic research (CPGR). Sequencing of samples using the NextSeq 500/550 Mid Output Kit v2 was carried out using the NextSeq 500 sequencer instrument (Illumina). 

RNA-seq data was analysed using the online bioinformatics tool, DNA subway, an iPlant Collaborative (recently renamed Cyverse) [60] further described as Protocol 1. These results were then validated against another analysis conducted by the CPGR, which will be described as Protocol 2.

#### 4.4.1. Analysis Using Protocol 1

Reads from the RNA-seq experiment received from the CPGR were analysed using the DNA Subway‘s Green Line RNA-seq workflow, which incorporates the Tuxedo suite of protocols [61]. Paired end reads received from the CPGR were aligned to the *Zea mays* B73 AGPv2 genome. 

Reads were processed using the FastX toolkit; reads with lower quality scores (<20) were filtered out. Individual reads were aligned to the reference genome using TopHat version 2.0.11 to determine intron/exon boundaries, and expression levels were calculated using CuffDiff version 2.1.1 (*p* < 0.01) to determine differential expression between the control and infected samples (Table S1). 

#### 4.4.2. Analysis Using Protocol 2

The quality of the raw data for each input file was assessed using FastQC [62].

Raw sequencing reads were trimmed using Trimmomatic version 0.32 [63]. Adapters were clipped from the 3’ end of raw sequencing reads; low-quality ends from the reads were trimmed as well before continuing with any downstream analysis processes. The adapters were trimmed from the 3’ ends and bases were removed from the 5’ ends. The bases with a quality score of <30 was clipped from both the 3’ and 5’ ends. Reads were processed in a 5’ to 3’ direction with a final length of <50 bases.

Processed reads were aligned to the *Zea mays* B73 v3 reference genome using TopHat version 2.0.13 [21], which aligns the reads to the reference genome (Table S2). Transcripts were then assembled using Cufflinks [64]. Differentially expressed genes (DEGs) were determined by comparing gene expression levels in the infected samples compared to the control samples using Cuffdiff [61].

Jvenn [39], an integrative online tool for comparing lists, was used to create a Venn diagram showing the DEGs between the two protocols and the commonality between them.

### 4.5. Gene Ontology Analysis Using agriGO

Using the Gene IDs from the RNA-seq analysis, gene ontology was analysed using agriGO [40]. This was a single enrichment analysis (SEA) using the *Zea mays* AGPv3.30 genome as a reference. The statistical method used in agriGO was the Fisher’s Exact Test with multi-test adjustment (Yekutieli-FDR under dependency) with significance level set at *p* < 0.05.

### 4.6. cDNA Synthesis and Quantitative Real-Time PCR 

For RT-qPCR, a total of 1 µg of purified RNA was used for cDNA synthesis. Duplicate cDNA synthesis was carried out on RNA extracted from control and infected leaf samples. *Ubiquitin-conjugating enzyme (UBCE)*, *DNA directed RNA-polymerase (Rpol)* and Membrane protein PB1A10.07c (*MEP)* served as reference genes (RG). Gene expression by RT-qPCR was performed on six RNA samples from control (3) and infected (3) maize leaves. Amplification was performed using the Rotor-Gene 6000 Series software (Corbett Life Science Research, Sydney, Australia).

### 4.7. Phytoalexin Accumulation

Phytoalexin analysis was performed as previously described [23,43] using approximately 100 mg maize tissue of each replicate from non-infected and infected maize shoot tissue, respectively. Phytoalexins were detected using a GC/isobutene chemical ion mass spectrometry (CI-MS) and quantified based on U-^13^C-18:3 (Cambridge Isotope Laboratories, Inc., Tewksbury, MA, USA) as an internal standard The analysis was done at the Chemistry Research Unit, Center for Medical, Agricultural, and Veterinary Entomology, United States Department of Agriculture–Agricultural Research Service (USDA–ARS) in Gainesville. 

### 4.8. Antioxidant Assays

The antioxidant activity of glutathione reductase (GR) and catalase (CAT) was assayed using a spectrophotometric approach. Enzyme extractions and assays measuring enzyme activity were performed according to Bailly et al. [65] with minor modifications. Approximately 0.25 g of maize leaf tissue for control and infected plants, after 14 days post-infection, was ground in liquid nitrogen to a fine powder. Total protein concentration was measured according to Bradford’s method [66] using BSA as a standard (Quick Start^TM^ Bradford Protein Assay) and carried out as per manufacturer’s instructions at an absorbance of 595 nm.

#### 4.8.1. Catalase (CAT, EC 1.11.1.6) 

Catalase activity was measured according to Claiborne [67]; the reaction was carried out at 25 °C. CAT activity was measured every second for 5 min and expressed as µmol H_2_O_2_ catalysed (g protein.sec)^−1^, where a decrease in absorbance of H_2_O_2_ was observed at 240 nm.

#### 4.8.2. Glutathione Reductase (GR, EC 1.6.4.2) 

Glutathione reductase activity was determined according to Bailly et al. [65]; activity was measured at 25 °C every minute for a period of 20 min at 340 nm in a microplate by observing the rate of NADPH oxidation. GR activity was expressed as µmol NADPH oxidised (mg protein)^−1^. 

### 4.9. Statistical Analysis

All statistical tests and graphs presented were generated using GraphPad Prism software version 5 and 6 (GraphPad Software Incorporation, 1992–2007). Unpaired t-tests were performed for the antioxidant assay experiments. For RT-qPCR, relative quantification was determined using the GenEx software (MultiD, Sweden), which incorporates the Pfaffl [68] analysis method and Excel. Statistical analysis was conducted using unpaired, parametric t-tests performed on the Log2 transformed values obtained from the control and infected samples 2 weeks after infection on all DEGs used for analysis. For phytoalexin accumulation, unpaired t-test was performed on log_10_ transformed data.

## Figures and Tables

**Table 1:** Significantly up- and down-regulated gene matches (*q* < 0.01) between Protocol 1 and Protocol 2 after *F. verticillioides* infection as detected by RNA-seq ^1^.

Gene Stable ID	Average Log2 Fold-Change	Ensembl/NCBI Gene Description	^¥^ Enriched GO-Term
GRMZM2G049538	4.56	Acyclic sesquiterpene synthase/ent-Kaurene synthase B	GO: 0043167
			GO: 0046872
			GO: 0043169
GRMZM2G119975	3.98	Uncharacterised LOC103646336	
GRMZM2G029219	3.45	Carbohydrate transporter/sugar porter/transporter	
GRMZM5G874955	3.33	Uncharacterised protein	
GRMZM2G062724	3.27	Uncharacterised protein	GO: 0043167
			GO: 0046872
			GO: 0043169
GRMZM2G026922	3.27	Hypothetical protein	
GRMZM2G117971	3.25	Uncharacterised protein	
GRMZM2G087875	3.20	Putative cytochrome P450 superfamily protein; Uncharacterised protein	GO: 0043167
			GO: 0046872
			GO: 0043169
GRMZM2G143139	3.17	N/A	
GRMZM2G149422	3.07	Hypothetical protein	
GRMZM2G154523	3.06	Patatin T5; Uncharacterised protein	
GRMZM2G098346	3.04	Alcohol dehydrogenase 2	GO: 0043167
			GO: 0046872
			GO: 0043169
GRMZM5G892675	3.01	Uncharacterised protein	
GRMZM2G137861	2.99	Wall-associated receptor kinase 2-like	
GRMZM2G443728	2.95	Potassium transporter 10	
GRMZM2G070011	2.85	Uncharacterised protein; Vignain	
GRMZM2G006973	2.73	Uncharacterised protein	GO: 0043167
			GO: 0046872
			GO: 0043169
GRMZM2G036464	2.72	Glutamine synthetase root isozyme 4	
GRMZM2G115451	2.63	Uncharacterised protein	
GRMZM2G178546	2.61	Trehalose-phosphate phosphatase	
GRMZM2G099049	2.56	N/A	
GRMZM2G063431	2.53	N/A	
GRMZM2G427815	2.51	Uncharacterised protein	GO: 0043167
			GO: 0046872
			GO: 0043169
GRMZM2G062531	2.50	Uncharacterised protein	GO: 0043167
			GO: 0046872
			GO: 0043169
GRMZM2G093826	2.49	Potassium high-affinity transporter	
GRMZM2G110504	2.46	Uncharacterised LOC100278648	
GRMZM2G007151	2.42	Uncharacterised protein	
GRMZM2G130173	2.41	Metallothionein-like protein type 2; Uncharacterised protein	GO: 0043167
			GO: 0046872
			GO: 0043169
GRMZM2G477503	2.36	Uncharacterised protein	
AC217947.4_FG002.2	2.24	N/A	GO: 0043167
			GO: 0046872
			GO: 0043169
GRMZM2G026470	2.22	Soluble inorganic pyrophosphatase; Uncharacterised protein	GO: 0043167
			GO: 0046872
			GO: 0043169
GRMZM2G091456	2.21	Putative Uncharacterised protein	
GRMZM2G366681	2.11	Hypothetical protein	
GRMZM2G034152	2.07	Polyamine oxidase	
GRMZM2G130149	2.07	Uncharacterised protein	
GRMZM2G144097	2.04	Uncharacterised protein	
GRMZM2G125669	2.03	Alternative oxidase	GO: 0043167
			GO: 0046872
			GO: 0043169
GRMZM2G144083	2.02	Putative ATP dependent copper transporter	GO: 0043167
			GO: 0046872
			GO: 0043169
GRMZM2G034302	2.01	Uncharacterised protein	
GRMZM2G036217	1.91	Uncharacterised protein	
GRMZM2G116079	1.86	Uncharacterised protein	GO: 0043167
			GO: 0046872
			GO: 0043169
GRMZM2G076537	1.82	Polynucleotidyl transferase, ribonuclease H-like superfamily protein	
GRMZM2G176433	1.80	Putative Uncharacterised protein	
GRMZM2G008247	1.78	Beta-glucosidase2	
GRMZM2G070659	1.77	Hypersensitive-induced response protein	
GRMZM2G147243	1.72	IAA17-auxin-responsive Aux/IAA family member; Uncharacterised protein	
GRMZM2G099767	1.63	ATMAP70-2	
GRMZM2G057823	1.55	Fructose-bisphosphate aldolase, cytoplasmic isozyme	GO: 0043167
			GO: 0046872
			GO: 0043169
GRMZM2G040369	1.54	Elongation factor 2	GO: 0043167
			GO: 0046872
			GO: 0043169
GRMZM2G168552	1.53	Bundle sheath cell specific protein 1	
GRMZM2G020146	1.51	Uncharacterised protein	
GRMZM2G473001	1.48	Phosphoenolpyruvate carboxylase 2	
GRMZM2G113332	1.42	Uncharacterised protein	GO: 0043167
			GO: 0046872
			GO: 0043169
GRMZM2G141353	1.41	Uncharacterised LOC100194210	
GRMZM2G146004	−FC	Uncharacterised protein	GO: 0050896
AC214438.3_FG002.1	−FC	N/A	
GRMZM2G177077	−1.40	Glucose-6-phosphate 1-dehydrogenase	GO: 0005975
GRMZM2G047474	−1.42	TLD-domain containing nucleolar protein	
GRMZM2G366659	−1.49	Putative trehalose phosphatase/synthase family protein	GO: 0005975
GRMZM5G870170	−1.57	MATE1	GO: 0050896
			GO: 0042221
GRMZM2G024733	−1.57	Uncharacterised LOC100304285	
GRMZM2G478568	−1.63	Nicotianamine synthase 3	
GRMZM2G154278	−1.68	Pre-mRNA-splicing factor cwc15	
GRMZM2G103812	−1.70	Uncharacterised protein	GO: 0050896
			GO: 0042221
GRMZM2G121264	−1.74	Uncharacterised protein	
GRMZM2G173085	−1.85	Lipase/lipooxygenase, PLAT/LH2 family protein	
GRMZM2G079381	−1.86	Ferredoxin-nitrite reductase, chloroplastic	GO: 0050896
			GO: 0042221
GRMZM2G053669	−1.99	Asparagine synthetase	
GRMZM2G147687	−2.02	Uncharacterised protein	GO: 0005975
GRMZM2G358153	−2.09	Chitinase 1; Uncharacterised protein	GO: 0005975
GRMZM2G181081	−2.21	CIPK-like protein 1	GO: 0050896
			GO: 0042221
GRMZM2G422955	−2.26	N/A	
GRMZM2G097641	−2.27	Sucrose-phosphatase 2	GO: 0005975
			GO: 0050896
			GO: 0042221
GRMZM2G078472	−2.32	Asparagine synthetase	
GRMZM2G124495	−2.38	Putative MYB DNA-binding domain superfamily protein; Transfactor; Uncharacterised protein	
GRMZM2G058612	−2.39	F-box/LRR-repeat protein 3-like	
GRMZM2G125775	−2.40	AN17	GO: 0050896
			GO: 0042221
GRMZM2G133675	−2.65	Putative HLH DNA-binding domain superfamily protein; Uncharacterised protein	
GRMZM2G004161	−2.92	Uncharacterised protein	GO: 0050896
			GO: 0042221
GRMZM2G472248	−3.17	Protein induced upon tuberization	GO: 0050896
GRMZM2G176430	−3.19	Uncharacterised protein	
GRMZM2G468111	−3.58	Uncharacterised LOC100277849	
GRMZM2G070172	−3.79	Uncharacterised protein	GO: 0005975
			GO: 0050896
			GO: 0042221

^1^ RNA-seq analysis using the Tuxedo suite and mapping to the maize B73 genome. Table shows annotation of genes as described in Plant Ensembl, NCBI with associated enriched GO-terms. [See Supplementary Tables for alternative gene names according to Blast2GO analysis]. The average Log2 fold-change is shown in a heatmap format with up-regulated genes in yellow and down-regulated genes in blue. FC: Gene is only expressed (down- regulated) in one experimental group and not the other. **^¥^** GO = Gene ontology, discussed in next section.

**Table 2:** Significant GO terms from matching up-regulated and down-regulated genes resulting from the agriGO database search using *Zea mays* AGPv3.30 as the reference genome.

Up-Regulated Genes						
GO Term	Ontology ^1^	Description	Number in Input List	Number in BG/Ref	*p*-Value ^2^	FDR
GO:0043169	F	cation binding	16 /45	4135/25864	0.0011 *	0.028
GO:0043167	F	ion binding	16 /45	4136/25864	0.0011 *	0.028
GO:0046872	F	metal ion binding	16 /45	4124/25864	0.0011 *	0.028
**Down-regulated genes**						
GO:0050896	P	response to stimulus	10/22	3551/25864	0.00032 *	0.0059
GO:0042221	P	response to chemical stimulus	8/22	2052/25864	0.00018 *	0.0059
GO:0005975	P	carbohydrate metabolic process	6/22	1246/25864	0.00048 *	0.0060

^1^ Gene ontology: F = molecular function, P = biological process; ^2^ For statistical analysis, Fisher’s Exact Test was performed with multi-test adjustment and a cut-off of 0.05 after performing a SEA. Asterisk indicates statistically significant *p*-values.

**Table 3:** Up-regulated genes from the matching DEG list found to be specific to the susceptible/resistant genotype or common to both genotypes from the Lanubile et al. study [22].

Gene Stable ID	Ensembl/NCBI Gene Description	Susceptible/Resistant/Common ^1^	Functional Category ^2^
GRMZM2G125669	Alternative oxidase	Common	Response to stress
GRMZM2G093826	Potassium high-affinity transporter	Common	Transport
GRMZM5G874955	Uncharacterised protein	Common	Transport
GRMZM2G029219	Carbohydrate transporter/sugar porter/transporter	Common	Transport
GRMZM2G036464	Glutamine synthetase root isozyme 4	Common	Metabolic process
GRMZM2G008247	Beta-glucosidase 2	Common	Cell wall
GRMZM2G034152	Polyamine oxidase	Common	Metabolic process
GRMZM2G427815	Uncharacterised protein	Common	Resistance
GRMZM5G892675	Uncharacterised protein	Common	Transport
GRMZM2G087875	Putative cytochrome P450 superfamily protein	Common	Metabolic process
GRMZM2G020146	Uncharacterised protein	Resistant	Metabolic process
GRMZM2G130173	Metallothionein-like protein type 2; Uncharacterised protein	Resistant	Unknown function
GRMZM2G026470	Soluble inorganic pyrophosphatase	Resistant	Metabolic process
GRMZM2G062724	Uncharacterised protein	Resistant	Signal transduction
GRMZM2G178546	Trehalose-phosphate phosphatase	Susceptible	Metabolic process
GRMZM2G007151	Uncharacterised protein	Susceptible	Cell component
GRMZM2G119975	Uncharacterised LOC103646336	Susceptible	Metabolic process

^1^ Differentially expressed genes found in the susceptible (CO354), resistant (CO441) as well as commonly expressed (found in the susceptible and resistant genotype) as found in the Lanubile et al., study [22]. ^2^ Differentially expressed genes belonging to their specific functional categories as per Blast2GO (found in the Lanubile et al., study [22]).

**Table 4:** Metabolite accumulation of A and B kauralexins in control and *F. verticillioides*-infected maize shoot tissue 2 weeks after infection.

Metabolite	^¥^ Logged Average Metabolite Accumulation		Standard Deviation	*p*-Value (Control/Infected)
	Control	Infected		
KA1	1.42	2.07	0.59	0.09
KA2	1.68	1.60	1.11	0.90
KA3	1.90	2.89	0.67	0.03 *
KB1	1.68	2.60	0.54	0.02 *
KB2	1.60	2.51	0.90	0.11
KB3	1.78	3.06	0.93	0.04 *

^¥^ Log transformed data shown above (mean, significant * *p* < 0.05, *n* = 4, two tailed Students t-test on log transformed data). KA refers to the class A kauralexins (1–3) and KB refers to the class B kauralexins (1–3).

## Supplementary Materials

The following are available online at https://www.mdpi.com/2223-7747/9/9/1112/s1. References [69,70,71,72] are cited in the supplementary materials: Figure S1: Graph showing singular enrichment analysis (SEA) results obtained using the agriGO database for Gene Ontology analysis of up-regulated genes between Protocol 1 and Protocol 2 gene matches. The blue bars represent the GO term enrichment for the input gene list and the green bars represent the enrichment for the background/reference genome (*Zea mays* AGPv3.30). Figure S2: Graph showing singular enrichment analysis (SEA) results obtained using the agriGO database for Gene Ontology analysis of down-regulated genes between Protocol 1 and Protocol 2 gene matches. The blue bars represent the GO term enrichment for the input gene list and the green bars represent the enrichment for the background/reference genome (Zea mays AGPv3.30). Figure S3: Quantitative real-time PCR analysis of average gene expression of ZmAn2 (GRMZM2G044481) and ZmKSL4 (GRMZM2G016922) genes in *F. verticillioides* infected shoot tissue relative to control shoot tissue 14-days post inoculation. Expression was normalised to the appropriate reference genes (Table S2) and shown as relative values (Log2-fold change). The RT-qPCR results were compared against average RNA-seq analysis results (mean of Protocol 1 and Protocol to Log2-fold values). Error bars indicate standard error of the mean (SEM). Table S1: Number of reads from the control and infected samples successfully mapped to the Zea mays B73 v2 reference genome and overall mapping rate (%) of the reads using Protocol 1 for RNA-seq analysis. Table S2: Number of reads from the control and infected samples successfully mapped to the Zea mays B73 v3 reference genome and overall mapping rate (%) of the reads using Protocol 2 for RNA-seq analysis. Table S3: In planta fungal quantification of fungal DNA (relative to plant DNA) in two-week old plants. Table S4: List of primers used for validation of RNA-seq data by RT-qPCR on control and infected maize. Table S5: Significantly up-regulated genes from Protocol 1 after F. verticillioides infection as detected using RNA-seq with the tuxedo suite of analysis and mapping to the maize B73 v3 genome. Table shows annotation of genes as described in Plant Ensembl, NCBI and Maize Microarray Annotation Database (Blast2GO). Table S6: Significantly down-regulated genes of Protocol 1 after *F. verticillioides* infection as detected using RNA-seq with the Tuxedo suite of analysis and mapping to the maize B73 v3 genome. Table shows annotation of genes as described in Plant Ensembl, NCBI and Maize microarray annotation database (Blast2GO). Table S7: Significantly up-regulated genes from Protocol 2 after *F. verticillioides* infection as detected using RNA-seq with the Tuxedo suite of analysis and mapping to the maize B73 v3 genome. Table shows annotation of genes as described in Plant Ensembl, NCBI and Maize microarray annotation database (Blast2GO). Table S8: Significantly down-regulated genes of Protocol 2 after *F. verticillioides* infection as detected using RNA-seq with the Tuxedo suite of analysis and mapping to the maize B73 v3 genome. Table shows annotation of genes as described in Plant Ensembl, NCBI and Maize microarray annotation database (Blast2GO).
