# Supplementary material for: Transcriptomic Analysis of a Susceptible African Maize Line to Fusarium verticillioides Infection
Source: Plants (Basel). 2020 Aug 28;9(9):1112. doi: 10.3390/plants9091112 (PMC7569872; doi:10.3390/plants9091112)
Supplement: Supplementary file 1 [file plants-09-01112-s001.pdf]

plants

SUPPLEMENTARY MATERIAL

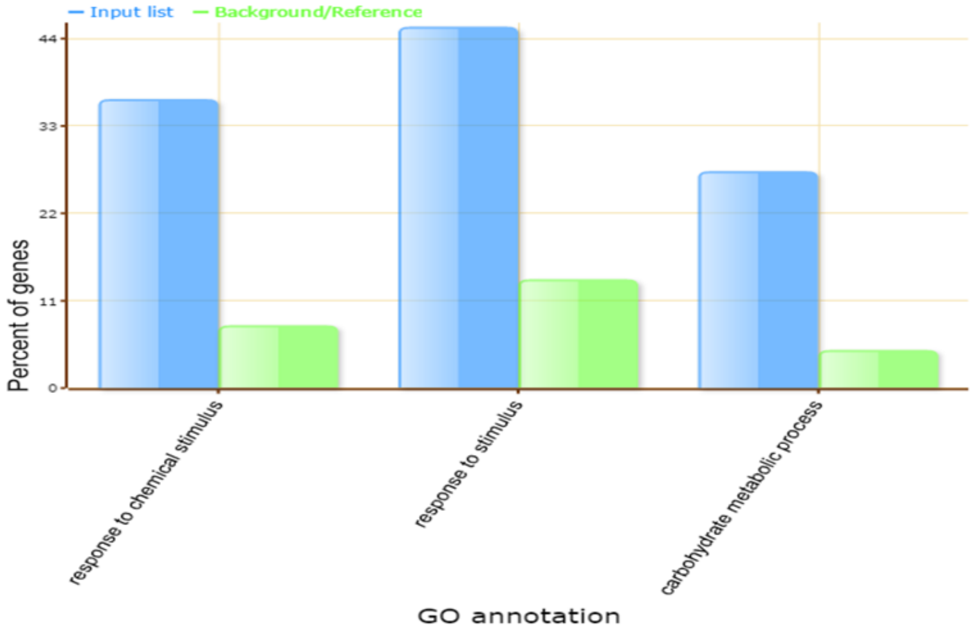

Figure 1. Graph showing singular enrichment analysis (SEA) results obtained using the agriGO database for Gene Ontology analysis of up-regulated genes between Protocol 1 and Protocol 2 gene matches. The blue bars represent the GO term enrichment for the input gene list and the green bars represent the enrichment for the background/reference genome (*Zea mays* AGPv3.30).

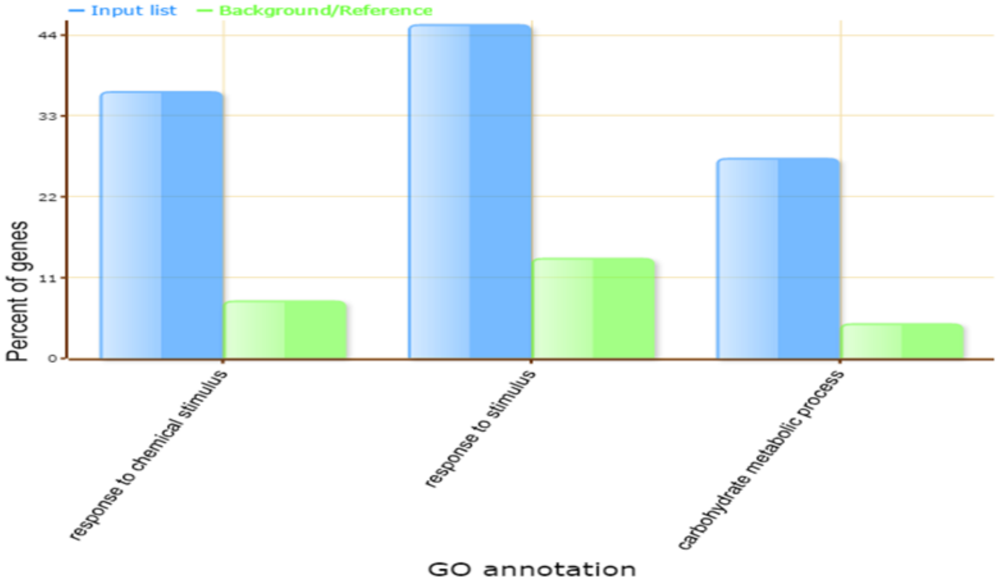

Figure 2. Graph showing singular enrichment analysis (SEA) results obtained using the agriGO database for Gene Ontology analysis of down-regulated genes between Protocol 1 and Protocol 2 gene matches. The blue bars represent the GO term enrichment for the input gene list and the green bars represent the enrichment for the background/reference genome (*Zea mays* AGPv3.30).

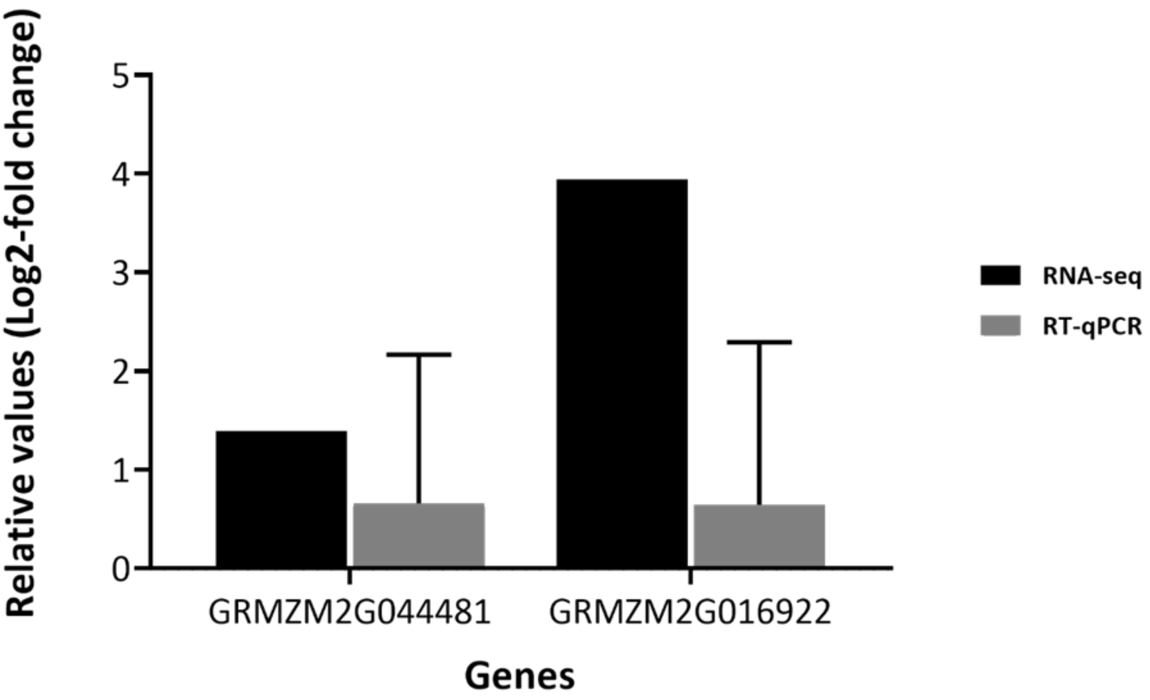

Figure 3. Quantitative real-time PCR analysis of average gene expression of *ZmAn2* (GRMZM2G044481) and *ZmKSL4* (GRMZM2G016922) genes in *F. verticillioides* infected shoot tissue relative to control shoot tissue 14-days post inoculation. Expression was normalised to the appropriate reference genes (Table S2) and shown as relative values (Log2-fold change). The RT-qPCR results were compared against average RNA-seq analysis results (mean of Protocol 1 and Protocol to Log2-fold values). Error bars indicate standard error of the mean (SEM).

Table S1. Number of reads from the control and infected samples successfully mapped to the *Zea mays* B73 v2 reference genome and overall mapping rate (%) of the reads using Protocol 1 for RNA-seq analysis.

Sample	No. of Mapped Reads		Overall Mapping Rate (%)
	Left	Right	
**Control 1**	10293607	11066641	62.6
**Control 2**	13072640	14726719	66.7
**Control 3**	11102335	12638559	67.4
**Infected 1**	9757579	10159711	62.6
**Infected 2**	10542335	11764997	65.9
**Infected 3**	12159647	14237213	70.3

Table S2. Number of reads from the control and infected samples successfully mapped to the *Zea mays* B73 v3 reference genome and overall mapping rate (%) of the reads using Protocol 2 for RNA-seq analysis.

Sample	No. of Mapped Reads		Overall Mapping Rate (%)
	Left	Right	
Control 1	10355561	11133885	63.0
Control 2	13131727	14801185	67.0
Control 3	11157732	12705814	67.8
Infected 1	9819472	10218423	63.0
Infected 2	10567766	11790914	66.1
Infected 3	12226508	14312632	70.8

Table S3. In planta fungal quantification of fungal DNA (relative to plant DNA) in two-week old plants.

Fungal quantification	Biological replicate: Fungal DNA/Plant DNA (ng.µg^{-1})				Average fungal DNA/Plant DNA (ng.µg^{-1})	Standard deviation	*p*-value: infected/control
	1	2	3	4			
Control	0	0	0	0	0	0	
Infected	1.575	0.468	9.126	1.015	3.05	3.53	0.23

Quantification of EF1α/MEP was measured by RT-qPCR in the shoots of control and infected plants (mean + std; $n = 4$). Fungal DNA (ng) was calculated as a ratio to plant DNA (µg) with significance $p < 0.05$ using a two-tailed Student's t-test.

Table S4. List of primers used for validation of RNA-seq data by RT-qPCR on control and infected maize.

Gene Name	Gene Product	Primer Sequences (Forward (F)/Reverse (R)) --> 5'-3'	Amplicon Size (bp)	Annealing Temperature (T_A) °C	Reference
**Reference genes**					
UBCE	*Ubiquitin-conjugating enzyme*	**F:**TGCGTTAATCACGAGACAGG<br>**R:**AATCACAAAGACAGGCAGGG	267	60	Manoli et al., 2012 [69]
Rpol	*DNA directed RNA-polymerase*	**F**:AGCCAAAACGCTAAAGTGGA<br>**R**:TAAGTGACGAGCAAGGCAAA	175	58	Ma et al., 2006 [70]
MEP	*Membrane protein PB1A10.07c*	**F:**TGTACTCGGCAATGCTCTTG<br>**R:**TTTGATGCTCCAGGCTTACC	203	60	Manoli et al., 2012 [69]
**Gene of interest (post-validation)**					
PR-th	*Pathogenesis related-thaumatin-like protein* **(GRMZM2G039639)**	**F:**GGGGTAATTCGGAGCAGC<br>**R:**ACGAGCGGGAAGAGGTG	72	60	This study
Chitinase 1	*Chitinase 1* **(GRMZM2G358153)**	**F:**GGGCTGTTCATCTGGTCG<br>**R:**GATCTGCTGCGCCTCGGT	78	60	This study
HIR3	*Hypersensitive induced reaction 3* **(GRMZM2G070659)**	**F:**GGGAGGCAGAAGCCAAGT<br>**R:**GACGGAGAACCCCAGCAC	98	58	This study
PIT	*Protein induced upon tuberization* **(GRMZM2G472248)**	**F:**GCGAACGGCGTGTTGAGC<br>**R:**CGCACCGAGAAGACAGAA	100	58	This study
lox6	*Lipoxygenase 6* **(GRMZM2G040095)**	**F:**GCCCGCCGGAAAACTGCA<br>**R:**CTCGTAGGCGATGCTCCC	123	60	This study
ZmCYP81A1	Putative cytochrome P450 **(GRMZM2G087875)**	**F:** TTTCAGCTCATCGCACGCTG<br>**R:** CGTCAAGAGGTGGTGGAGCGAGC	119	60	Veenstr, 2017 [71]
ZmTPS1	ent-Kaurene synthase B/ terpene synthase 1 **(GRMZM2G049538)**	**F:** TAACGCAAGCCCAAGAGAGC<br>**R:** AGGAACTAAGCGTCGATGTGATG	163	60	Modified from Schnee et al., 2002 [72]

Table S5. Significantly up-regulated genes from Protocol 1 after *F. verticillioides* infection as detected using RNA-seq with the tuxedo suite of analysis and mapping to the maize B73 v3 genome. Table shows annotation of genes as described in Plant Ensembl, NCBI and Maize Microarray Annotation Database (Blast2GO). .

Key- Bold: Protocol 1 vs. Protocol 2 matches; Lanubile *et al*., [22] genes: Susceptible genotype , Resistant genotype ;.

qRT-PCR genes: ; (*): Sequencing in progress; FC/-FC: Gene is only expressed (up-/down- regulated) in one experimental group and not the other.

Chr	Gene Stable ID	log2 (fold-change)	*p*-value	*q*-value	Ensembl/ NCBI gene description	Blast2GO description
1	GRMZM2G062724	3.19	5.00E-05	0.006114	Uncharacterised protein	chy zinc finger family expressed
	GRMZM2G034302	1.72	5.00E-05	0.006114	Uncharacterised protein	sucrose transporter
	GRMZM2G130149	2.10	5.00E-05	0.006114	Uncharacterised protein	myb family transcription expressed
	GRMZM2G137861	3.13	5.00E-05	0.006114	Wall-associated receptor kinase 2-like	N/A
	GRMZM2G099767	1.66	5.00E-05	0.006114	ATMAP70-2	N/A
	GRMZM2G091456	2.19	5.00E-05	0.006114	Putative Uncharacterised protein	squalene expressed, squalene monooxygenase
	GRMZM2G366681	2.11	5.00E-05	0.006114	Hypothetical protein	N/A
	GRMZM2G040369	1.57	5.00E-05	0.006114	Elongation factor 2	protein
	GRMZM2G076537	1.86	5.00E-05	0.006114	Polynucleotidyl transferase, ribonuclease H-like superfamily protein	exonuclease family protein
	GRMZM2G061817	1.68	5.00E-05	0.006114	N/A	N/A
	GRMZM2G576460	2.78	5.00E-05	0.006114	N/A	cysteine proteinase
	GRMZM2G162879	2.42	5.00E-05	0.006114	N/A	N/A
2	GRMZM2G049538	4.71	5.00E-05	0.006114	Acyclic sesquiterpene synthase	ent-kaurene synthase b
	GRMZM2G125669	1.97	5.00E-	0.006	Alternative oxidase	alternative oxidase

			5.00E-05	0.006114		
	GRMZM2G115451	2.63	5.00E-05	0.006114	Uncharacterised protein	neutral alkaline invertase
	GRMZM2G144083	2.06	5.00E-05	0.006114	Putative ATP dependent copper transporter	heavy metal p-type atpase
	GRMZM2G176433	1.84	5.00E-05	0.006114	Putative Uncharacterised protein	N/A
	GRMZM2G119975	3.90	5.00E-05	0.006114	Uncharacterised LOC103646336	N/A
	GRMZM2G093826	2.29	5.00E-05	0.006114	Potassium high-affinity transporter	high-affinity potassium transporter
	GRMZM2G587803	1.53	5.00E-05	0.006114	N/A	N/A
	GRMZM2G394450	2.15	5.00E-05	0.006114	Beta-fructofuranosidase 1	beta-fructofuranosidase 1
3	GRMZM2G062531	2.38	5.00E-05	0.006114	Uncharacterised protein	c-4 sterol methyl oxidase
	GRMZM2G057823	1.48	5.00E-05	0.006114	Fructose-bisphosphate aldolase, cytoplasmic isozyme	fructose-bisphosphate aldolase
	GRMZM5G874955	3.40	5.00E-05	0.006114	Uncharacterised protein	pdr-like abc transporter
	GRMZM2G143139	3.28	5.00E-05	0.006114	N/A	N/A
	GRMZM2G029219	3.52	5.00E-05	0.006114	Carbohydrate transporter/ sugar porter/ transporter	major facilitator superfamily antiporter/ carbohydrate transporter sugar porter transporter
	GRMZM2G122296	2.84	5.00E-05	0.006114	Putative phosphoethanolamine N-methyltransferase	phosphoethanolamine n-methyltransferase
	GRMZM5G896496	1.62	5.00E-05	0.006114	N/A	heat shock protein 93-v
	GRMZM2G161310	1.62	5.00E-05	0.006114	Uncharacterised protein	carbohydrate transporter sugar porter transporter
	GRMZM2G050275	2.53	5.00E-05	0.006114	N/A	N/A
4	GRMZM2G098346	2.96	5.00E-05	0.006114	Alcohol dehydrogenase 2	alcohol dehydrogenase

	GRMZM2G026922	3.28	5.00E-05	0.006114	Hypothetical protein	acetylglutamate kinase
	GRMZM2G070011	2.81	5.00E-05	0.006114	Uncharacterised protein; Vignain	vignain precursor
	GRMZM2G036464	2.64	5.00E-05	0.006114	Glutamine synthetase root isozyme 4	glutamine synthetase
	GRMZM2G149422	2.91	5.00E-05	0.006114	Hypothetical protein	phi-1
	GRMZM2G116079	1.80	5.00E-05	0.006114	Uncharacterised protein	N/A
	GRMZM2G036217	1.85	5.00E-05	0.006114	Uncharacterised protein	fatty acyl coa reductase
	GRMZM2G154523	3.10	5.00E-05	0.006114	Patatin T5; Uncharacterised protein	patatin t5 recursor*
	AC217947.4_FG002	2.23	5.00E-05	0.006114	NADPH-cytochrome P450 reductase	N/A
	GRMZM2G117971	3.39	5.00E-05	0.006114	Uncharacterised protein	pathogenesis-related protein 4
	GRMZM2G165387	2.08	5.00E-05	0.006114	Uncharacterised protein	leucine rich repeat family expressed
	GRMZM2G018424	1.60	5.00E-05	0.006114	N/A	N/A
	GRMZM2G075430	1.55	5.00E-05	0.006114	N/A	N/A
	GRMZM2G122076	1.44	5.00E-05	0.006114	Homeodomain leucine zipper protein CPHB-5; Putative homeobox DNA-binding and leucine zipper domain family protein	homeodomain leucine zipper protein cphb-5
	GRMZM5G864911	1.55	5.00E-05	0.006114	N/A	N/A
5	GRMZM2G113332	1.37	5.00E-05	0.006114	Uncharacterised protein	copper chaperone
	GRMZM2G144097	2.07	5.00E-05	0.006114	Uncharacterised protein	protein
	GRMZM2G020146	1.50	5.00E-05	0.006114	Uncharacterised protein	serine carboxypeptidase iii precursor
	GRMZM2G134903	1.63	5.00E-05	0.006114	Exonuclease; Uncharacterised protein	exonuclease family protein
6	GRMZM2G147243	1.68	5.00E-	0.006	IAA17-auxin-responsive Aux/IAA family member; Uncharacterised protein	transcription factor

			05	114		
	GRMZM2G070659	1.65	5.00E-05	0.006114	Hypersensitive-induced response protein	hypersensitive-induced response protein
	GRMZM2G130173	2.38	5.00E-05	0.006114	Metallothionein-like protein type 2; Uncharacterised protein	N/A
	GRMZM2G088501	1.97	5.00E-05	0.006114	Uncharacterised LOC100193404	N/A
	GRMZM2G158972	1.52	5.00E-05	0.006114	Putative inositol polyphosphate phosphatase (Synaptogenin-like) family protein	ipsi (inositol polyphosphate 5-phosphatase i) inositol triphosphate phosphatas inositol-polyphosphate 5-phosphatase
7	GRMZM2G099049	2.68	5.00E-05	0.006114	N/A	N/A
	GRMZM2G427815	2.44	5.00E-05	0.006114	Uncharacterised protein	peroxidase
	GRMZM2G473001	1.45	5.00E-05	0.006114	Phosphoenolpyruvate carboxylase 2	phosphoenolpyruvate carboxylase
	GRMZM2G149916	2.25	5.00E-05	0.006114	N/A	N/A
8	GRMZM2G026470	2.09	5.00E-05	0.006114	Soluble inorganic pyrophosphatase; Uncharacterised protein	soluble inorganic pyrophosphatase
	GRMZM2G477503	2.35	5.00E-05	0.006114	Uncharacterised protein	sulfolipid synthase
	GRMZM5G892675	3.12	5.00E-05	0.006114	Uncharacterised protein	N/A
	GRMZM2G063431	2.61	5.00E-05	0.006114	N/A	N/A
	GRMZM2G168552	1.56	5.00E-05	0.006114	Bundle sheath cell specific protein 1	N/A
	GRMZM2G087875	3.12	5.00E-05	0.006114	Putative cytochrome P450 superfamily protein; Uncharacterised protein	cytochrome p450 family expressed
	GRMZM2G007151	2.27	5.00E-05	0.006114	Uncharacterised protein	endomembrane-associated protein
	GRMZM2G110504	2.41	5.00E-05	0.006114	Uncharacterised LOC100278648	hypothetical protein LOC100278648 [*Zea mays*]
	GRMZM2G141353	1.36	5.00E-05	0.006114	Uncharacterised LOC100194210	N/A
9	GRMZM2G178546	2.71	5.00E-05	0.006114	Trehalose-phosphate phosphatase	N/A

	GRMZM2G006973	2.78	5.00E-05	0.006114	Uncharacterised protein	N/A
	GRMZM2G443728	2.97	5.00E-05	0.006114	Potassium transporter 10	potassium transporter 10
	GRMZM2G116966	1.33	5.00E-05	0.006114	Benzoate carboxyl methyltransferase	N/A
10	GRMZM2G008247	1.73	5.00E-05	0.006114	Beta-glucosidase 2	N/A
	GRMZM2G034152	2.00	5.00E-05	0.006114	Polyamine oxidase	polyamine oxidase precursor
	GRMZM2G006490	1.91	5.00E-05	0.006114	N/A	N/A
	GRMZM2G173413	3.44	5.00E-05	0.006114	allantoinase [Source:Projected from Arabidopsis thaliana (AT4G04955) TAIR;Acc:AT4G04955]	protein

Table 6. Significantly down-regulated genes of Protocol 1 after *F. verticillioides* infection as detected using RNA-seq with the Tuxedo suite of analysis and mapping to the maize B73 v3 genome. Table shows annotation of genes as described in Plant Ensembl, NCBI and Maize microarray annotation database (Blast2GO)

**Chr**	**Gene stable ID**	**log2 (fold-change)**	***p*-value**	***q*-value**	**Ensembl/ NCBI gene description**	**Blast2GO description**
1	GRMZM2G070172	-3.88	5.00E-05	0.006114	Uncharacterised protein	N/A
	GRMZM2G478568	-1.62	5.00E-05	0.006114	Nicotianamine synthase 3	nicotianamine synthase 3
	GRMZM2G147687	-1.89	5.00E-05	0.006114	Uncharacterised protein	glycosyl hydrolase family 3 n terminal domain containing expressed
	GRMZM2G053669	-2.07	5.00E-05	0.006114	Asparagine synthetase	asparagine synthetase
	GRMZM5G815358	-1.80	5.00E-05	0.006114	Uncharacterised LOC100274071	N/A
	GRMZM2G106928	-2.52	5.00E-05	0.006114	Superoxide dismutase [Cu-Zn]	copper zinc superoxide dismutase
	GRMZM5G857674	-1.60	5.00E-05	0.006114	N/A	N/A
	GRMZM2G066441	-1.49	5.00E-05	0.006114	Uncharacterised protein	N/A

2	GRMZM2G1 25775	-2.36	5.00E -05	0.006 114	AN17	arsenite inducuble rna associated protein aip-701
	GRMZM2G0 47474	-1.49	5.00E -05	0.006 114	TLD-domain containing nucleolar protein	protein
	GRMZM2G4 99582	-FC	5.00E -05	0.006 114	N/A	N/A
	GRMZM2G3 48956	-1.39	5.00E -05	0.006 114	DNA mismatch repair protein MutS, type 2	2 family protein
	GRMZM2G0 78480	-1.65	5.00E -05	0.006 114	Cinnamoyl-CoA reductase 2-like	N/A
	GRMZM2G1 21264	-1.70	5.00E -05	0.006 114	Uncharacterised protein	cytochrome p450
3	GRMZM2G1 03812	-1.66	5.00E -05	0.006 114	Uncharacterised protein	selenium-binding protein
	GRMZM2G0 04161	-3.03	5.00E -05	0.006 114	Uncharacterised protein	btb and taz domain protein
	GRMZM2G4 68111	-3.46	5.00E -05	0.006 114	Uncharacterised LOC100277849	N/A
	GRMZM2G1 81081	-2.27	5.00E -05	0.006 114	CIPK-like protein 1	cipk-like protein expressed
	GRMZM2G4 72248	-3.23	5.00E -05	0.006 114	Protein induced upon tuberization	N/A
	GRMZM2G0 24733	-1.60	5.00E -05	0.006 114	Uncharacterised LOC100304285	pq-loop repeat family protein
	GRMZM2G4 79423	-1.50	5.00E -05	0.006 114	Aldose reductase	aldose reductase
	GRMZM5G8 14451	-1.40	5.00E -05	0.006 114	N/A	3-cyclic-nucleotide phosphodiesterase rega
	GRMZM2G1 02572	-1.70	5.00E -05	0.006 114	Isoamyl acetate-hydrolyzing esterase	isoamyl acetate-hydrolyzing
4	GRMZM2G3 58153	-2.02	5.00E -05	0.006 114	Chitinase 1; Uncharacterised protein	chitinase 1
	GRMZM2G1 73085	-1.86	5.00E -05	0.006 114	Lipase/lipooxygenase, PLAT/LH2 family protein	potential zinc finger protein
	GRMZM2G0 79381	-1.92	5.00E -05	0.006 114	Ferredoxin--nitrite reductase, chloroplastic	nitrite reductase
*	AC214438.3_	-FC	5.00E	0.006	N/A	N/A

	FG002		-05	114		
	GRMZM2G133675	-2.74	5.00E-05	0.006114	Putative HLH DNA-binding domain superfamily protein; Uncharacterised protein	amelogenin precursor like protein
	GRMZM2G366659	-1.49	5.00E-05	0.006114	Putative trehalose phosphatase/synthase family protein	trehalose 6-phosphate synthase
	GRMZM2G161198	-FC	5.00E-05	0.006114	N/A	N/A
	GRMZM2G157522	-1.57	5.00E-05	0.006114	Hypothetical protein LOC103654120	N/A
	GRMZM2G033236	-1.85	5.00E-05	0.006114	N/A	ubiquitin-activating enzyme
	GRMZM2G326911	-FC	5.00E-05	0.006114	N/A	N/A
	GRMZM2G570968	-FC	5.00E-05	0.006114	N/A	N/A
	GRMZM2G377695	-FC	5.00E-05	0.006114	N/A	N/A
5	GRMZM2G171539	-FC	5.00E-05	0.006114	N/A	mitochondrial nadh ubiquinone oxidoreductase 13kd-like subunit
6	GRMZM5G873765	-2.84	5.00E-05	0.006114	N/A	N/A
	GRMZM2G093325	-1.37	5.00E-05	0.006114	CONTAINS InterPro DOMAIN/s: Sgf11, transcriptional regulation (InterPro:IPR013246)	N/A
	GRMZM5G870170	-1.59	5.00E-05	0.006114	MATE1	mate efflux family protein
7	GRMZM2G176430	-3.35	5.00E-05	0.006114	Uncharacterised protein	sodium-dicarboxylate cotransporter
	GRMZM2G422955	-2.28	5.00E-05	0.006114	N/A	N/A
	GRMZM2G099879	-FC	5.00E-05	0.006114	N/A	N/A
8	GRMZM2G519073	-1.58	5.00E-05	0.006114	Uncharacterised protein	slt1 protein
	GRMZM2G154278	-FC	5.00E-05	0.006114	Pre-mRNA-splicing factor cwc15	N/A
	GRMZM2G146004	-FC	5.00E-05	0.006114	Uncharacterised protein	N/A

9	GRMZM2G078472	-2.44	5.00E-05	0.006114	Asparagine synthetase	asparagine synthetase
	GRMZM2G042510	-1.79	5.00E-05	0.006114	N/A	N/A
	GRMZM2G152417	-1.96	5.00E-05	0.006114	AMP-binding protein; Putative AMP-dependent synthetase and ligase superfamily protein; Uncharacterised protein	amp dependent
10	GRMZM2G124495	-2.52	5.00E-05	0.006114	Putative MYB DNA-binding domain superfamily protein; Transfactor; Uncharacterised protein	N/A
	GRMZM2G058612	-2.40	5.00E-05	0.006114	F-box/LRR-repeat protein 3-like	N/A
	GRMZM2G097641	-2.25	5.00E-05	0.006114	Sucrose-phosphatase 2	sucrose phosphate synthase
	GRMZM2G177077	-1.40	5.00E-05	0.006114	Glucose-6-phosphate 1-dehydrogenase	glucose-6-phosphate 1-dehydrogenase
	GRMZM2G152135	-2.12	5.00E-05	0.006114	Beta-carotene hydroxylase 1	beta-carotene hydroxylase 1

Table S7. Significantly up-regulated genes from Protocol 2 after *F. verticillioides* infection as detected using RNA-seq with the Tuxedo suite of analysis and mapping to the maize B73 v3 genome. Table shows annotation of genes as described in Plant Ensembl, NCBI and Maize microarray annotation database (Blast2GO).

Chr	Gene Stable ID	log2 (fold-change)	*p*-value	*q*-value	Ensembl / NCBI Gene Description	Blast2GO Description
1	GRMZM2G062724	3.35	5.00E-05	0.004255	Uncharacterised protein	chy zinc finger family expressed
	GRMZM2G034302	2.30	5.00E-05	0.004255	Uncharacterised protein	sucrose transporter
	GRMZM2G130149	2.04	5.00E-05	0.004255	Uncharacterised protein	myb family transcription expressed
	GRMZM2G137861	2.84	5.00E-05	0.004255	Wall-associated receptor kinase 2-like	N/A
	GRMZM2G456217	2.82	5.00E-05	0.004255	Vignain	cysteine proteinase
	GRMZM2G161274	2.15	5.00E-05	0.004255	Ribonuclease 3; Uncharacterised protein	s-like rnase
	GRMZM2G073725	1.73	5.00E-05	0.004255	Alpha-1,4-glucan-protein synthase [UDP-forming]	reversibly glycosylated polypeptide
	GRMZM2G099767	1.61	5.00E-05	0.004255	ATMAP70-2	N/A
	GRMZM2G091456	2.23	5.00E-05	0.004255	Putative Uncharacterised protein	squalene expressed, squalene monooxygenase
	GRMZM2G119755	FC	5.00E-05	0.004255	Cell number regulator 7	N/A
	GRMZM2G366681	2.12	5.00E-05	0.004255	Hypothetical protein	N/A
	GRMZM2G040369	1.52	5.00E-05	0.004255	Elongation factor 2	protein
	GRMZM2G076537	1.79	5.00E-05	0.004255	Polynucleotidyl transferase, ribonuclease H-like superfamily protein	exonuclease family protein
2	GRMZM2G049538	4.40	5.00E-05	0.004255	Acyclic sesquiterpene synthase	ent-kaurene synthase b
	GRMZM2G125669	2.10	5.00E-05	0.004255	Alternative oxidase	alternative oxidase
	GRMZM2G115451	2.64	5.00E-05	0.004255	Uncharacterised protein	neutral alkaline invertase
	GRMZM2G144083	1.99	0.0001	0.007870	Putative ATP dependent copper transporter	heavy metal p-type atpase
	GRMZM2G062156	2.03	5.00E-05	0.004255	Uncharacterised protein	N/A
	GRMZM2G176433	1.75	5.00E-05	0.004255	Putative Uncharacterised protein	N/A
	GRMZM2G119975	4.07	5.00E-05	0.004255	Uncharacterised LOC103646336	N/A
	GRMZM2G040095	3.21	5.00E-05	0.004255	Lipoxygenase	lipoxygenase
	GRMZM2G093826	2.68	5.00E-05	0.004255	Potassium high-affinity transporter	high-affinity potassium transporter
	GRMZM2G1	1.34	5.00E-0	0.00425	Uncharacterised protein	N/A

	06413		5	5	LOC100282066 / wound induced protein	
3	GRMZM2G062531	2.62	5.00E-05	0.004255	Uncharacterised protein	c-4 sterol methyl oxidase
	GRMZM2G057823	1.63	5.00E-05	0.004255	Fructose-bisphosphate aldolase, cytoplasmic isozyme	fructose-bisphosphate aldolase
	GRMZM2G022915	2.89	0.0001	0.007870	N/A	N/A
	GRMZM5G874955	3.26	5.00E-05	0.004255	Uncharacterised protein	pdr-like abc transporter
	GRMZM2G143139	3.05	5.00E-05	0.004255	N/A	N/A
	GRMZM2G402977	FC	0.0001	0.007870	N/A	N/A
	GRMZM2G029219	3.38	5.00E-05	0.004255	Carbohydrate transporter/ sugar porter/ transporter	major facilitator superfamily antiporter/ carbohydrate transporter sugar porter transporter
	GRMZM2G141665	1.67	5.00E-05	0.004255	Uncharacterised protein	syringomycin biosynthesis enzyme
4	GRMZM2G098346	3.12	5.00E-05	0.004255	Alcohol dehydrogenase 2	alcohol dehydrogenase
	GRMZM2G026922	3.26	5.00E-05	0.004255	Hypothetical protein	acetylglutamate kinase
	GRMZM2G070011	2.89	5.00E-05	0.004255	Uncharacterised protein; Vignain	vignain precursor
	GRMZM2G036464	2.79	5.00E-05	0.004255	Glutamine synthetase root isozyme 4	glutamine synthetase
	GRMZM2G047319	FC	5.00E-05	0.004255	Putative subtilase family protein	N/A
	GRMZM2G149422	3.22	5.00E-05	0.004255	Hypothetical protein	phi-1
	GRMZM2G041699	2.47	5.00E-05	0.004255	Cytokinin-O-glucosyltransferase 2	cytokinin-o-glucosyltransferase 2
	GRMZM2G343828	FC	5.00E-05	0.004255	Putative O-Glycosyl hydrolase superfamily protein	N/A
	GRMZM2G015295	1.60	5.00E-05	0.004255	Adenosylhomocysteinase	s-adenosyl-l-homocysteine hydrolase
	GRMZM2G116079	1.92	5.00E-05	0.004255	Uncharacterised protein	N/A
	GRMZM2G015419	2.01	5.00E-05	0.004255	Uncharacterised protein	lipoxygenase, lipoxygenase chloroplast precursor
	GRMZM2G036217	1.96	5.00E-05	0.004255	Uncharacterised protein	fatty acyl coa reductase
	GRMZM2G154523	3.02	5.00E-05	0.004255	Patatin T5; Uncharacterised protein	N/A
*	AC217947.4_FG002.2	2.25	0.0001	0.007870	N/A	N/A
	GRMZM2G117971	3.11	5.00E-05	0.004255	Uncharacterised protein	pathogenesis-related protein 4
5	GRMZM2G094353	3.54	5.00E-05	0.004255	Uncharacterised protein	rna-binding protein cabeza
	GRMZM2G0	2.30	5.00E-0	0.00425	N/A	N/A

	38874		5	5		
	GRMZM2G113332	1.47	5.00E-05	0.004255	Uncharacterised protein	copper chaperone
	GRMZM2G165530	2.04	0.0001	0.007870	Putative Uncharacterised protein	tetracycline transporter
	GRMZM2G144097	2.00	0.0001	0.007870	Uncharacterised protein	protein
*	AC225718.2_FG004	FC	5.00E-05	0.004255	N/A	N/A
	GRMZM2G060659	1.67	5.00E-05	0.004255	Putative Uncharacterised protein	protein
	GRMZM2G173192	FC	5.00E-05	0.004255	Uncharacterised protein	N/A
	GRMZM2G020146	1.53	5.00E-05	0.004255	Uncharacterised protein	serine carboxypeptidase iii precursor
	GRMZM2G130053	3.30	5.00E-05	0.004255	Cysteine protease 1	N/A
	GRMZM2G011888	1.96	5.00E-05	0.004255	Putative Uncharacterised protein	N/A
	GRMZM2G075333	3.01	5.00E-05	0.004255	Uncharacterised protein	4-coumarate: ligase
6	GRMZM2G036564	1.59	5.00E-05	0.004255	Transmembrane protein20	embryogenesis transmembrane
	GRMZM2G147243	1.76	5.00E-05	0.004255	IAA17-auxin-responsive Aux/IAA family member; Uncharacterised protein	transcription factor
	GRMZM2G124799	FC	5.00E-05	0.004255	Uncharacterised protein	N/A
	GRMZM2G070659	1.89	5.00E-05	0.004255	Hypersensitive-induced response protein	hypersensitive-induced response protein
	GRMZM5G844094	2.44	5.00E-05	0.004255	N/A	N/A
	GRMZM2G130173	2.44	5.00E-05	0.004255	Metallothionein-like protein type 2; Uncharacterised protein	N/A
	GRMZM2G100719	FC	0.00005	0.004255	N/A	N/A
7	GRMZM2G099049	2.44	5.00E-05	0.004255	N/A	N/A
	GRMZM2G003179	1.81	5.00E-05	0.004255	copper transporter 5	N/A
	GRMZM2G086714	1.90	0.0001	0.007870	Uncharacterised LOC103632825	plastid ppgpp synthase
	GRMZM2G050172	1.90	0.0001	0.007870	Uncharacterised LOC103632825	plastid ppgpp synthase
	GRMZM5G884407	2.07	5.00E-05	0.004255	N/A	aldehyde dehydrogenase
	GRMZM5G817559	2.65	5.00E-05	0.004255	Uncharacterised protein	protein
	GRMZM2G415529	2.33	5.00E-05	0.004255	N/A	pdr-like abc transporter
	GRMZM2G366977	FC	5.00E-05	0.004255	Equilibrative nucleotide transporter 3-like	N/A
	GRMZM2G427815	2.59	5.00E-05	0.004255	Uncharacterised protein	peroxidase
	GRMZM2G170734	FC	5.00E-05	0.004255	Chlorophyllase-2, chloroplastic-like	N/A
	GRMZM2G473001	1.51	5.00E-05	0.004255	Phosphoenolpyruvate carboxylase 2	phosphoenolpyruvate

						carboxylase
8	GRMZM2G026470	2.36	5.00E-05	0.004255	Soluble inorganic pyrophosphatase; Uncharacterised protein	soluble inorganic pyrophosphatase
	GRMZM6G198866	1.68	5.00E-05	0.004255	Metallothionein-like protein type 2	N/A
	GRMZM2G070912	1.68	5.00E-05	0.004255	Putative metallothionein family protein	N/A
	GRMZM2G477503	2.36	5.00E-05	0.004255	Uncharacterised protein	sulfolipid synthase
	GRMZM5G892675	2.90	5.00E-05	0.004255	Uncharacterised protein	N/A
	GRMZM2G063431	2.46	5.00E-05	0.004255	N/A	N/A
	GRMZM2G077054	1.37	5.00E-05	0.004255	Uncharacterised protein	nadh-dependent glutamate synthase 1 gene
	GRMZM2G173718	1.40	5.00E-05	0.004255	Uncharacterised LOC100273627	N/A
	GRMZM2G455124	FC	5.00E-05	0.004255	nitrate transporter2.	N/A
	GRMZM2G168552	1.51	5.00E-05	0.004255	Bundle sheath cell specific protein 1	N/A
	GRMZM5G875238	1.42	0.0001	0.007870	Sucrose-phosphate synthase	sucrose phosphate synthase
	GRMZM2G022958	1.92	5.00E-05	0.004255	Uncharacterised LOC100275172	N/A
	GRMZM2G087875	3.28	5.00E-05	0.004255	Putative cytochrome P450 superfamily protein; Uncharacterised protein	cytochrome p450 family expressed
	GRMZM2G007151	2.57	5.00E-05	0.004255	Uncharacterised protein	endomembrane-associated protein
	GRMZM2G054123	3.62	5.00E-05	0.004255	S-adenosylmethionine synthase	N/A
	GRMZM2G097141	5.20	5.00E-05	0.004255	N/A	N/A
	GRMZM2G110504	2.51	5.00E-05	0.004255	Uncharacterised LOC100278648	hypothetical protein LOC100278648 [Zea mays]
	GRMZM2G141353	1.45	5.00E-05	0.004255	Uncharacterised LOC100194210	N/A
9	GRMZM2G178546	2.51	5.00E-05	0.004255	Trehalose-phosphate phosphatase	N/A
	GRMZM2G132238	2.13	5.00E-05	0.004255	Putative metacaspase family protein	N/A
	GRMZM2G479243	7.08	5.00E-05	0.004255	Putative leucine-rich repeat receptor-like protein kinase family protein	brassinosteroid insensitive 1-associated receptor kinase 1
	GRMZM2G006973	2.68	5.00E-05	0.004255	Uncharacterised protein	N/A
	GRMZM2G035285	3.66	0.0001	0.007870	N/A	N/A
	GRMZM2G443728	2.94	5.00E-05	0.004255	Potassium transporter 10	potassium transporter 10
10	GRMZM2G147390	FC	5.00E-05	0.004255	Uncharacterised protein	N/A

	GRMZM2G034882	1.87	5.00E-05	0.004255	Uncharacterised LOC100276570	N/A
	GRMZM2G008247	1.82	5.00E-05	0.004255	Beta-glucosidase2	N/A
	GRMZM2G034152	2.14	5.00E-05	0.004255	Polyamine oxidase	polyamine oxidase precursor
	GRMZM2G163998	1.85	0.0001	0.007870	Uncharacterised protein; VAMP protein SEC22	N/A
scaffold_510:0-2226	GRMZM6G761998	1.89	5.00E-05	0.004255	Zinc transporter 2	N/A

Table 8. Significantly down-regulated genes of Protocol 2 after *F. verticillioides* infection as detected using RNA-seq with the Tuxedo suite of analysis and mapping to the maize B73 v3 genome. Table shows annotation of genes as described in Plant Ensembl, NCBI and Maize microarray annotation database (Blast2GO).

Chr	Gene Stable ID	log2 (fold-change)	*p*-value	*q*-value	Ensembl/NCBI Gene Description	Blast2GO Description
1	GRMZM2G070685	-1.86	5.00E-05	0.004255	N/A	N/A
	GRMZM2G001877	-1.34	5.00E-05	0.004255	N/A	N/A
	GRMZM2G070172	-4.06	5.00E-05	0.004255	Uncharacterised protein	N/A
	GRMZM2G039639	-1.83	5.00E-05	0.004255	Protein P21	pathogenesis-related thaumatin-like protein
	GRMZM2G478568	-1.63	5.00E-05	0.004255	Nicotianamine synthase 3	nicotianamine synthase 3
	GRMZM2G147687	-2.15	5.00E-05	0.004255	Uncharacterised protein	glycosyl hydrolase family 3 n terminal domain containing expressed
	GRMZM2G061626	-FC	0.0001	0.007870	ZFP16-2	
	GRMZM2G053669	-1.92	5.00E-05	0.004255	Asparagine synthetase	asparagine synthetase
2	GRMZM2G125775	-2.44	5.00E-05	0.004255	AN17	arsenite inducuble rna associated protein aip-701
	GRMZM2G121264	-1.78	5.00E-05	0.004255	Uncharacterised protein	cytochrome p450
	GRMZM2G047474	-1.35	5.00E-05	0.004255	TLD-domain containing nucleolar protein	protein
	GRMZM2G015024	-1.34	5.00E-05	0.004255	50S ribosomal protein L22, chloroplastic	N/A
3	GRMZM2G103812	-1.73	5.00E-05	0.004255	Uncharacterised protein	selenium-binding protein
	GRMZM2G166548	-2.09	5.00E-05	0.004255	N/A	N/A
	GRMZM2G004161	-2.80	5.00E-05	0.004255	Uncharacterised protein	N/A
	GRMZM2G468111	-3.71	5.00E-05	0.004255	Uncharacterised LOC100277849	N/A
	GRMZM2G181081	-2.14	5.00E-05	0.004255	CIPK-like protein 1	cipk-like protein expressed
*	AC194022.3_FG013.1	-2.76	5.00E-05	0.004255	N/A	N/A
	GRMZM2G472248	-3.11	5.00E-05	0.004255	Protein induced upon tuberization	N/A
	GRMZM2G024733	-1.54	5.00E-05	0.004255	Uncharacterised LOC100304285	pq-loop repeat family protein
4	GRMZM2G358153	-2.15	5.00E-05	0.004255	Chitinase 1; Uncharacterised protein	chitinase 1
	GRMZM5G845532	-FC	0.0001	0.007870		
	GRMZM2G173085	-1.85	5.00E-05	0.004255	Lipase/lipooxygenase, PLAT/LH2 family protein	potential zinc finger protein

	GRMZM2G079381	-1.79	5.00E-05	0.004255	Ferredoxin--nitrite reductase, chloroplastic	nitrite reductase
	GRMZM5G863229	-1.77	5.00E-05	0.004255	Uncharacterised protein	protein
*	AC214438.3_FG002.1	-FC	5.00E-05	0.004255	N/A	N/A
	GRMZM2G133675	-2.56	5.00E-05	0.004255	Putative HLH DNA-binding domain superfamily protein; Uncharacterised protein	amelogenin precursor like protein
	GRMZM2G366659	-1.49	5.00E-05	0.004255	Putative trehalose phosphatase/synthase family protein	trehalose 6-phosphate synthase
	GRMZM2G061126	-1.35	5.00E-05	0.004255	Hypothetical protein	N/A
5	AC212351.4_FG001.1	-1.32	5.00E-05	0.004255	Uncharacterised LOC100502277	N/A
	GRMZM2G057766	-FC	5.00E-05	0.004255	Chitinase 1	N/A
	GRMZM5G878558	-3.61	5.00E-05	0.004255	Uncharacterised protein	nitrate reductase
	GRMZM2G083788	-2.00	0.0001	0.004255	Vacuolar amino acid transporter 1-like	amino acid transporter family protein
	GRMZM2G168747	-FC	5.00E-05	0.007870	Metal transporter NRAT1-like	N/A
6	GRMZM5G847462	-FC	5.00E-05	0.004255	N/A	N/A
	GRMZM5G870170	-1.54	5.00E-05	0.004255	MATE1	mate efflux family protein
7	GRMZM2G176430	-3.03	5.00E-05	0.004255	Uncharacterised protein	sodium-dicarboxylate cotransporter
	GRMZM2G422955	-2.24	5.00E-05	0.004255	N/A	N/A
	GRMZM2G016212	-FC	5.00E-05	0.004255	N/A	N/A
8	GRMZM2G020594	-4.97	5.00E-05	0.004255	F-box domain containing protein; F-box domain containing protein isoform 1; F-box domain containing protein isoform 2; Uncharacterised protein	N/A
	GRMZM2G154278	-3.36	5.00E-05	0.004255	Pre-mRNA-splicing factor cwc15	N/A
	GRMZM2G146004	-FC	5.00E-05	0.004255	Uncharacterised protein	N/A
9	GRMZM2G078472	-2.20	5.00E-05	0.004255	Asparagine synthetase	asparagine synthetase
10	GRMZM2G455476	-2.06	0.0001	0.004255	Uncharacterised protein	white-brown-complex abc transporter family
	GRMZM2G124495	-2.24	5.00E-05	0.007870	Putative MYB DNA-binding domain superfamily protein; Transfactor; Uncharacterised protein	N/A
	GRMZM2G058612	-2.39	5.00E-05	0.004255	F-box/LRR-repeat protein 3-like	N/A
	GRMZM2G064008	-FC	5.00E-05	0.004255	N/A	N/A
	GRMZM2G097641	-2.28	5.00E-05	0.004255	Sucrose-phosphatase 2	sucrose phosphate synthase
	GRMZM2G177077	-1.42	5.00E-05	0.004255	Glucose-6-phosphate 1-dehydrogenase	glucose-6-phosphate

						1-dehydrogenase
scaffold_509:407701-410547	GRMZM2G149326	-1.77	5.00E-05	0.004255	N/A	N/A
